# Optimizing mild surface cleaning methods: influence of water purity and pH

**DOI:** 10.1038/s41598-025-15143-0

**Published:** 2025-08-14

**Authors:** Andriani Tsompou, Vitaly Kocherbitov

**Affiliations:** 1 Department of Biomedical Science, Malmö University, Malmö, SE-20506 Sweden; 2 Biofilms research center for Biointerfaces, Malmö University, Malmö, Sweden

**Keywords:** Washing and cleaning, Surfactant-free, Alkalinity, Pure water, Olive oil, Contact angle, Surface chemistry, Colloids

## Abstract

Due to growing concerns about the environmental impact of detergents, there has been a notable shift towards researching eco-friendly washing methods, such as using purified water for washing and cleaning. It has been shown that purified water can remove olive oil from hydrophilic surfaces but removing it from hydrophobic surfaces is still a challenge. In this work we studied the removal of olive oil from hydrophobic and hydrophilic surfaces using different water alkalinity, different salt solutions, multiple washing cycles and temperatures. For the hydrophobic surface, gravimetric analysis data demonstrated that non-purified water grades can outperform purified ones, but this effect is due to slight variations of pH. Increasing the pH of purified water by addition of tiny amounts of NaOH (that would not have any environmental impact) significantly enhances cleaning efficiency. For the hydrophilic surfaces, water with increased alkalinity completely removes the oil from the surface in most cases. The study reveals that adjusted pH of otherwise pure water promotes deprotonation of fatty acids in olive oil and facilitates oil removal from surfaces through roll up and interfacial tension reduction mechanisms. Increased temperatures further improve cleaning efficiency. These findings highlight the potential of pH-adjusted purified water as an effective and eco-friendly alternative to conventional cleaning methods. Future research should explore similar techniques on complex materials such as textiles.

**Supplementary Information:**

The online version contains supplementary material available at 10.1038/s41598-025-15143-0.

## Introduction

In recent years, growing environmental concerns have increased the attention towards developing sustainable alternatives to synthetic detergents. Significant ecological dangers are associated with the extensive use of synthetic surfactants, which have been used for more than a century in industrial and domestic cleaning applications. More than 60% of surfactants produced globally end up in aquatic ecosystems, where they pollute water and negatively impact aquatic life^1–4^. Furthermore, synthetic detergents can harm people’s health since they leave residues on clothes or surfaces that have been cleaned, which can irritate skin and trigger allergic reactions^5–9^. To address these problems, research is focused on creating biodegradable, less toxic surfactants from natural sources that minimize environmental and health impacts^4,10–12^. Alongside this, alternative cleaning methods such as the use of ultra-pure water grades have been studied the last few years^13,14^. By removing or decreasing the amount of surfactant to low amounts, the process of washing is promoted by the properties of the purified water.

The effectiveness of ultra-pure water in removing hydrophobic soils from hydrophilic surfaces has been extensively studied, with key factors such as temperature and the number of washing cycles significantly influencing the cleaning process. This process is divided into two regimes: the first phase is dominated by surface mechanisms, including the “roll-up” of oil droplets supported by electrostatic repulsion, while the second phase involves bulk mechanisms, such as solubility, which become more prominent as the oil concentration decreases^13,14^. While oil removal using purified water on smooth hydrophilic surfaces is well understood, less attention has been given to more complex systems such as removing hydrophobic soils from hydrophobic surfaces.

Removing hydrophobic soils, such as olive oil, from hydrophobic surfaces is challenging due to several factors. The non-polar nature of both the soil and surface prevents interaction with water. Olive oil tends to spread over a hydrophobic surface, creating a thin film while forms droplets with contact angle values more than 90^o^^15,16^. Specifically for polypropylene (PP), the type of plastic that will be used in this research, the contact angle of water to the surface according to Young’s equations is 95^o^^16^. Although contact angle is a simple but straight forward phenomenon to separate hydrophilic from hydrophobic surfaces, physical and chemical heterogeneities complicate matters. One of these heterogeneities is the spreading coefficient. The spreading coefficient of water over a solid is the reduction in surface free energy when a solid/water and water/vapor interface is forming on the surface. For water on hydrophobic surfaces, the spreading coefficient will be negative^16^ as no spreading occurs. Because of this nonpolar nature, the removal of the oil from surfaces like PP, is not an easy task.

When surfactants are used for washing these surfaces, the hydrophobic tails are oriented towards the hydrophobic oil while the hydrophilic head groups will orient toward the water^17,18^. This adsorption of surfactants lowers the interfacial tension between the liquid and the hydrophobic surface. As a result, the contact angle between the liquid droplet and the hydrophobic surface decrease. However, due to strong nonpolar interactions between the soil and the surface, the surface is often not easily cleaned. It has been shown that non-ionic surfactants are more effective^18,19^ and increased mechanical energy is necessary to prevent re-deposition^19^.

To be able to promote an environmental way of washing and cleaning with purified water grades, many different systems should be studied and understood. After having studied the hydrophilic surfaces, it needs to be understood how hydrophobic surfaces can be washed with minimal use of surfactants. In our previous study^13^, it was found that washing a plastic surface with water grades with one cycle at 25 °C resulted in less than 20% washing efficiency^13^. This study aims to investigate the effectiveness of different water grades and pH levels in removing hydrophobic soils, specifically olive oil, from hydrophobic surfaces using gravimetric analysis. Based on these experimental results, we try to better understand the mechanism behind washing with purified water grades and how the detergency mechanisms work together in that system.

## Materials and methods

### Materials

For the following experiments, four different water grades and one solution were used, and they will be referred to as follows:

- MQ: Milli-Q Water purified using a Millipore Milli-Q lab water system. it is produced in the laboratory using PURELAB flex (ELGA, UK).
- DIRO: ultra-pure water provided and produced by SWATAB (Malmö, Sweden).
- ΤΑP: tap water from the university building, Malmö, Sweden. Further characterisation is presented in Table S1.
- NaCl: 10 mM NaCl solution in MQ water. Concentration will be specified if different than 10 mM.

MQ water was directly used for the experiment as the purification system is available in the laboratories. DIRO water was stored in 10 L plastic bottles while the NaCl solutions were stored in glass bottles.

Olive oil (extra virgin olive oil FONTANA est 1978, classic, Spain) was used as a hydrophobic soil. Polypropylene (PP) 15 mL plastic tubes (SARSTEDT, Germany) were used as the hydrophobic substrate. Sodium hydroxide, NaOH, was used for adjusting the pH of each water grade to the wanted value. Microscope glass slides (VWR, article No. 631–1551) were used as the hydrophilic substrate where contact angle measurements were performed. Atomic Force Microcopy (AFM) and Scanning Electron Microscopy (SEM) were performed to characterize the topology of the samples. Information can be found in Section 6 and 7 of the supplementary data.

Characterisation of the water grades can be found in our previous research^14^.

### Gravimetric analysis: measurement of oil film mass before and after water contact

To detect how the washing efficiency of plastic tubes is being affected by (i) water grades (ii) pH and (iii) different temperatures (iv) multiple washing cycles, the mass of the oil film on the tubes was measured before and after exposure to water under different conditions. The experiment was performed on 15 mL plastic tubes and after each step the mass of the tube (with the lid) was measured with a Mettler Toledo AT261 analytical scale. First, 14–15 mg of olive oil was placed on the tube surface and with the help of a cotton stick the oil was spread on the whole surface. 5 g of water was then added. The tubes were vortexed for 10 s and the excess water was discarded. The tubes were left to dry overnight in the freeze dryer ALPHA 1–4 LCS (temperature: −60 °C, pressure: 0.08 mbar) and their mass was measured the following day.

The temperatures of the water grades that were examined were 25, 40, and 60 °C and were controlled via a thermostat (LAUDA, ECO Gold, Germany). The number of washing cycles performed was between 1 and 11. The pH values that were studied varied between pH 7–11. MQ water was studied at five pH values while the other 3 grades were studied at two pH values (Table 1).

To prepare solutions with different pH values, a stock solution of 4.8 mM of NaOH was used. In the pH adjustment, the amounts of stock solution needed to achieve the target concentrations were added to water grades to get 500 ml pH-adjusted solutions. The pH values of the solutions were measured with a pH meter and are shown in Table 1.

**Table 1:** pH values and NaOH concentrations used for this study.

Water grade	pH	NaOH concentration (mM)
MQ	7.53	0.0001
MQ	7.98	0.001
MQ	8.99	0.01
MQ	10.01	0.1
MQ	11.02	1
DIRO	7.56	0.0001
NaCl	7.51	0.0001
TAP	7.98	0.0001
DIRO	11.1	1
NaCl	11.1	1
TAP	11.02	1

### Differential scanning calorimetry (DSC)

DSC measurements were performed using DSC 1 (Mettler Toledo, Switzerland). The temperature calibration and heat flow calibration were done using indium. All data analysis was done using STARe Software following the ISO standard (ISO 11357-2:1999). An empty aluminum crucible was used as a reference. The following protocol was used: equilibration at 25 °C for 5 min, heating 10 °C/min to 200 °C, isothermal for 5 min, cooling 10 °C/min to 25 °C. Two cycles were performed.

### Contact angle measurements

The Drop Shape Analysis System-DSA100S (Kruss, Germany) was used for the measurement of contact angle. A 5 µl oil droplet was placed at the middle of a glass surface. The glass was cleaned with ethanol, deionized water and dried with N2 gas. 3 mL of a water grade were added, and the contact angle was measured. The measurement of the contact angle was performed with the build-in software (ADVANCE) and the Young-Laplace equation was used for fitting. More than three samples were measured for each occasion. Mean values and standard deviations were calculated in MATLAB (The MathMorks, inc).

### Atomic force microscopy (AFM)

AFM (Multimode 8, Bruker) was used for topographic characterisation of glass slides. An AC240TS-R3 cantilever was used (Olympus cantilevers). Elastic constant was 1.42 N/m, calibrated using Sader’s method^20^. The sensitivity of the cantilever was 48.4 nm/V and was calibrated using thermal noise. Tapping mode was used as a measurement mode, with oscillation amplitude of 8 nm. All images were processed with flatten and equalise using WSxM^21^.

### Scanning electron microscopy (SEM)

Surface morphologies of the polypropylene plastic tubes were studied using a Zeiss EVO LS10 (Oberkochen, Germany) scanning electron microscope equipped with a LaB6 filament, operated at 25 °C in high vacuum using a secondary electron detector, at an acceleration voltage of 15 kV. Prior to measurements, the polypropylene pieces were fixed on pin-type aluminum stubs with the use of Leit-C conductive carbon cement (Agar Scientific) and sputter coated with a thin layer of gold using an Agar Automatic Sputter Coater at 30 mA, 0.08 mbar pressure and with a sputtering time of 40 s. A conducting silver bridge was painted from the top of the sample down to the stub using Electrodag 1415 conducting silver paint (Agar Scientific).

## Results and discussion

For this study, four different water grades were used to study their washing efficiency on hydrophobic surfaces. MQ and DIRO were the purified water grades while TAP water and NaCl solution represented the non-purified grades. The study examined how different parameters such as temperature, pH and salt content could influence the washing efficiency.

### Washing plastic tubes with different water grades

**Fig. 1:**
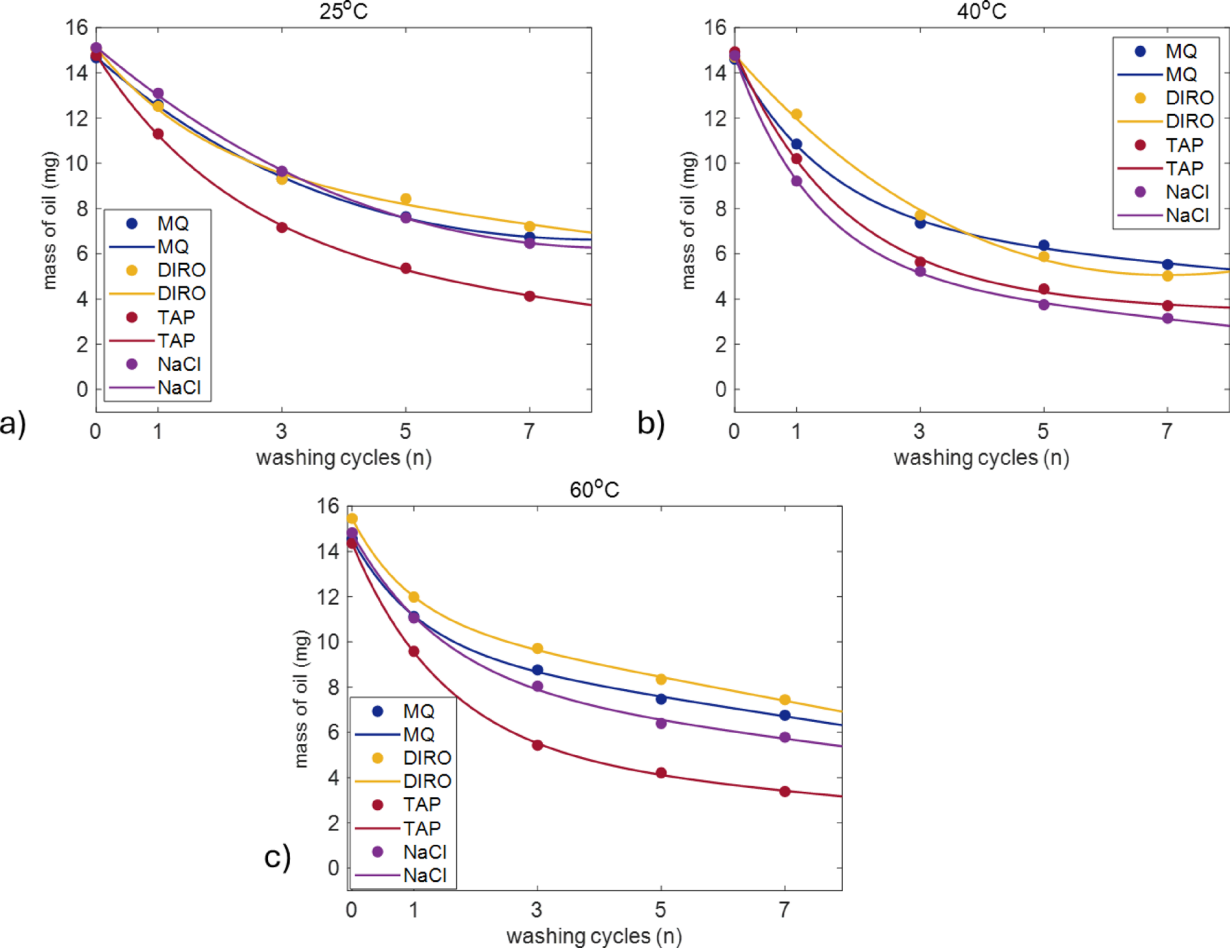
Average amount of olive oil left on the surface of plastic tubes (mg) as a function of the number of washing cycles for MQ, DIRO, NaCl, and TAP at **(a)** 25 °C **(b)** 40 °C and **(c)** 60 °C. The number of replicates is 3 for DIRO and NaCl and 6 for MQ and TAP. Plots with error bars can be found in the supplementary data (Figure S1).

Results of gravimetric measurements of washing at temperatures of 25, 40 and 60 °C with different water grades are shown in Fig. 1.

**Fig. 2:**
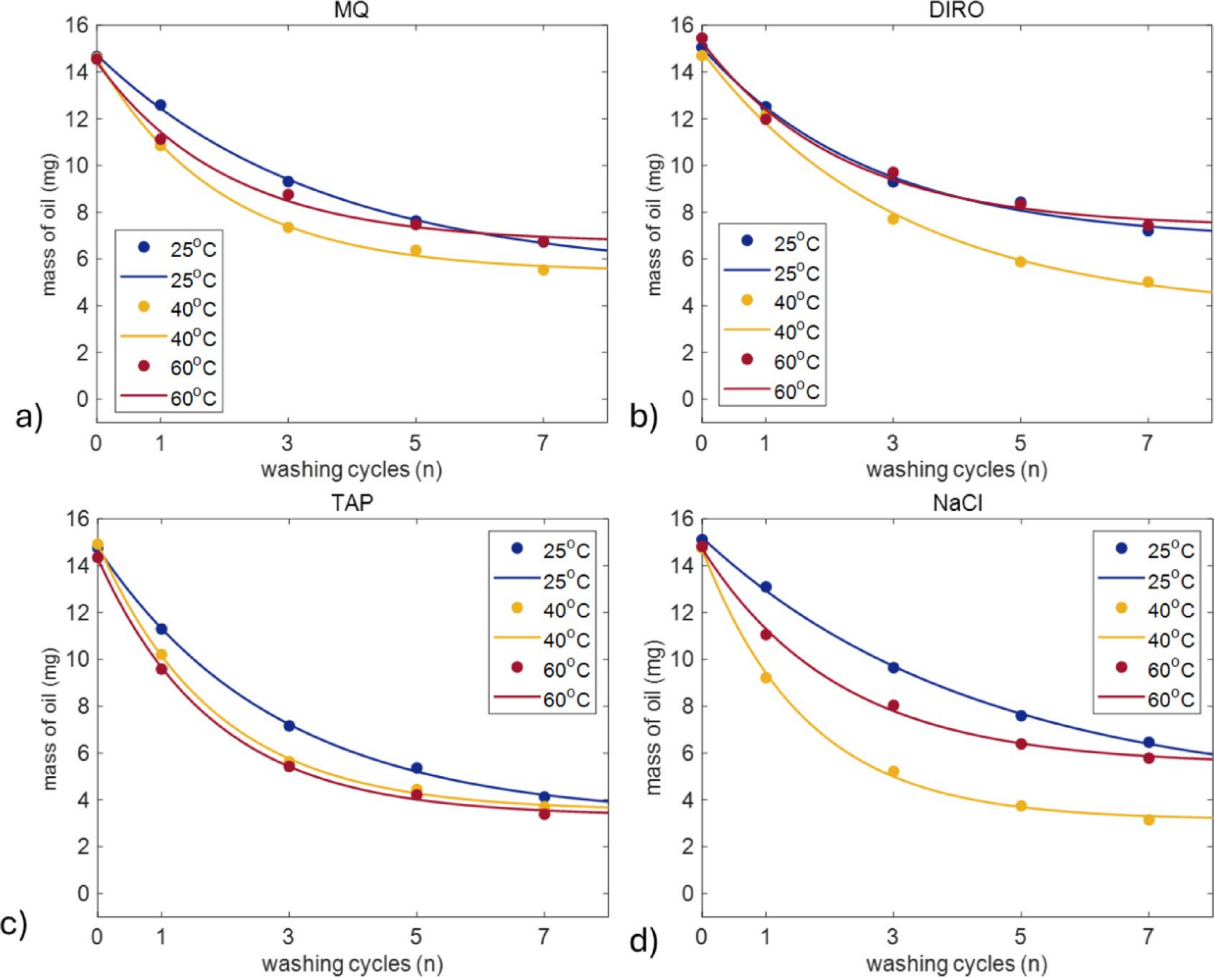
Average amount (mg) of oil left on the plastic surface after each washing cycle when different water grades **(a)** MQ **(b)** DIRO **(c)** TAP **(d)** NaCl were used at 25 °C, 40 °C, and at 60 °C. The number of replicates is 3 for DIRO and NaCl and 6 for MQ and TAP. Plots with error bars can be found in the supplementary data (Figure S2).

All the water grades show qualitatively the same behaviour. Most of the removed amount of oil was removed in the first three washing cycles while subsequent cycles showed a lower removal rate. Quantitative comparison, however, revealed a surprising result. The TAP water, in contrast to the results previously reported for glass surfaces, showed better efficiency than purified water grades at all three temperatures (Fig. 1). Although the error bars for efficiencies of different water grades overlap in some cases (Figure S1), the overall trend shows that TAP water has higher efficiency in cleaning the hydrophobic surface. To better analyse the effect of temperature on each water grade, the mass of remaining oil (mg) was replotted for each water grade individually as a function of the number of washing cycles across all temperatures (Fig. 2).

Figure 2 shows how the temperature influences the washing efficiency of each water grade. As expected, in most cases washing at 25 °C showed lowest efficiency. However, it seems that the temperature increase affects the washing efficiencies of water grades differently.

For purified water grades, 40 °C has the highest efficiency after 7 cycles (62% for MQ and 66% for DIRO). The efficiency is almost identical for 25 and 60 °C. For NaCl, the same behaviour is observed with 79% efficiency at 40 °C. For TAP water, higher temperatures (40 °C and 60 °C) resulted in faster oil removal compared to 25 °C. However, by the seventh cycle, the temperature difference becomes less pronounced. Figure S2 shows that the error bars show higher variability at the initial stages of washing, particularly at 25 °C, but the measurements become more consistent as the number of cycles increases.

The remaining oil mass showed an exponential decrease upon the number of washing cycles for all temperatures and water grades. Since in the later part of the curve one should expect a linear dependence, all experiments were fitted with the following equation^14^:

1 $$\:m=\:{m}^{o}-\:{m}_{s}^{o}\left(1-\:{e}^{-cn}\right)-an$$

were $\:m$ is the mass of removed oil (mg) after the washing cycle, $\:{m}^{o}$ is the initial mass of oil (mg), $\:{m}_{s}^{o}$ is the amount that can be removed during the exponential regime (mg), $\:c$ is the exponential decay constant, $\:a$ is the slope of the linear dependence and $\:n$ is the washing cycle number. As previously stated^14^ the slope *a* can be related to the apparent solubility, $\:{C}^{app}$ in mg/g, of the olive oil, as shown in Eq. (2).

2 $$\:{C}^{app}=\:\frac{a}{{m}_{t}}$$

were $\:{m}_{t}=5.015\:\text{g}$ is the total mass of the system (water + oil). The equation was first introduced in our previous work^14^, where the removal of oil on a glass surface exhibited an exponential and a linear behaviour of oil removal. In the current data, no linear behaviour is observed in the studied number of washing cycles hence the linear part was not used in the equation.

**Fig. 3:**
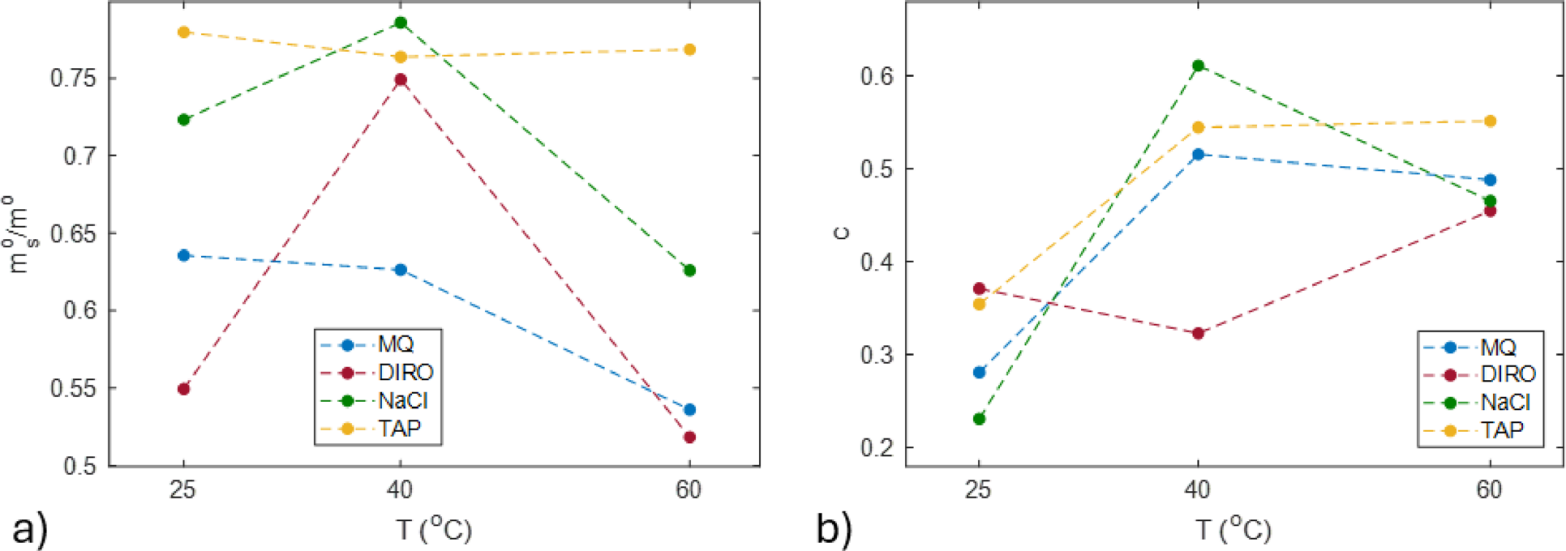
Parameters calculated from the exponential fit of gravimetric data obtained from plastic tubes for MQ, DIRO, NaCl and TAP at 25, 40 and 60 °C; **(a)** mass of oil removed during the exponential regime **(b)** exponential decay constant obtained using Eq. (1).

By plotting the parameters found in Eq. 1, it is found that both the amount of oil that is removed from the tubes and the oil decay is higher for NaCl and TAP (Fig. 3) in most of the cases. This agrees with the data observed above. Both the mass of oil removed and the exponential decay constant is higher for non-purified water grades (Fig. 3a). In almost all cases (at 25 and 60 °C), NaCl shows similar decay to the purified water grades. Interestingly, at 40 °C all water grades seem to have the best efficiency, with the non-purified grades still having the higher oil removal.

To understand why non-purified water grades generally exhibited better efficiency in oil removal, it is important to recognize the fundamental differences between water grades. Purified and non-purified water differ significantly in various factors, most notably in the presence of ions. While all water grades undergo multiple purification steps (mechanical, chemical, and biological)^22^, TAP water retains both monovalent ions (such as sodium) and divalent ions (such as magnesium and calcium), which are largely absent in ultra-pure water grades^23^. The concentration of these ions in TAP water depends on the water source and filtration methods used.

In our previous research, we observed that the removal of oily hydrophobic soils from hydrophilic surfaces is enhanced by water purity^13,14^. Specifically, the absence of ions in purified water increases electrostatic repulsion, which facilitates the detachment of oil from surfaces. As mentioned earlier, the oil removal in such systems can be divided into two distinct regimes. During the first, exponential, regime the amount of oil on the surface is high and surface mechanisms, such as roll-up, dominate. Oil droplets are kinetically stabilized in purified water by electrostatic interactions. The charge that exists on the surfaces in combination with the absence of ions in pure water leads to detachment of the soil and its stabilization in water. As the amount of oil on the surface decreases, the second, linear regime takes over, where bulk mechanisms become more relevant, and electrostatic interactions are less important^14^.

### Washing with na₂so₄ and cacl₂ solutions

**Fig. 4:**
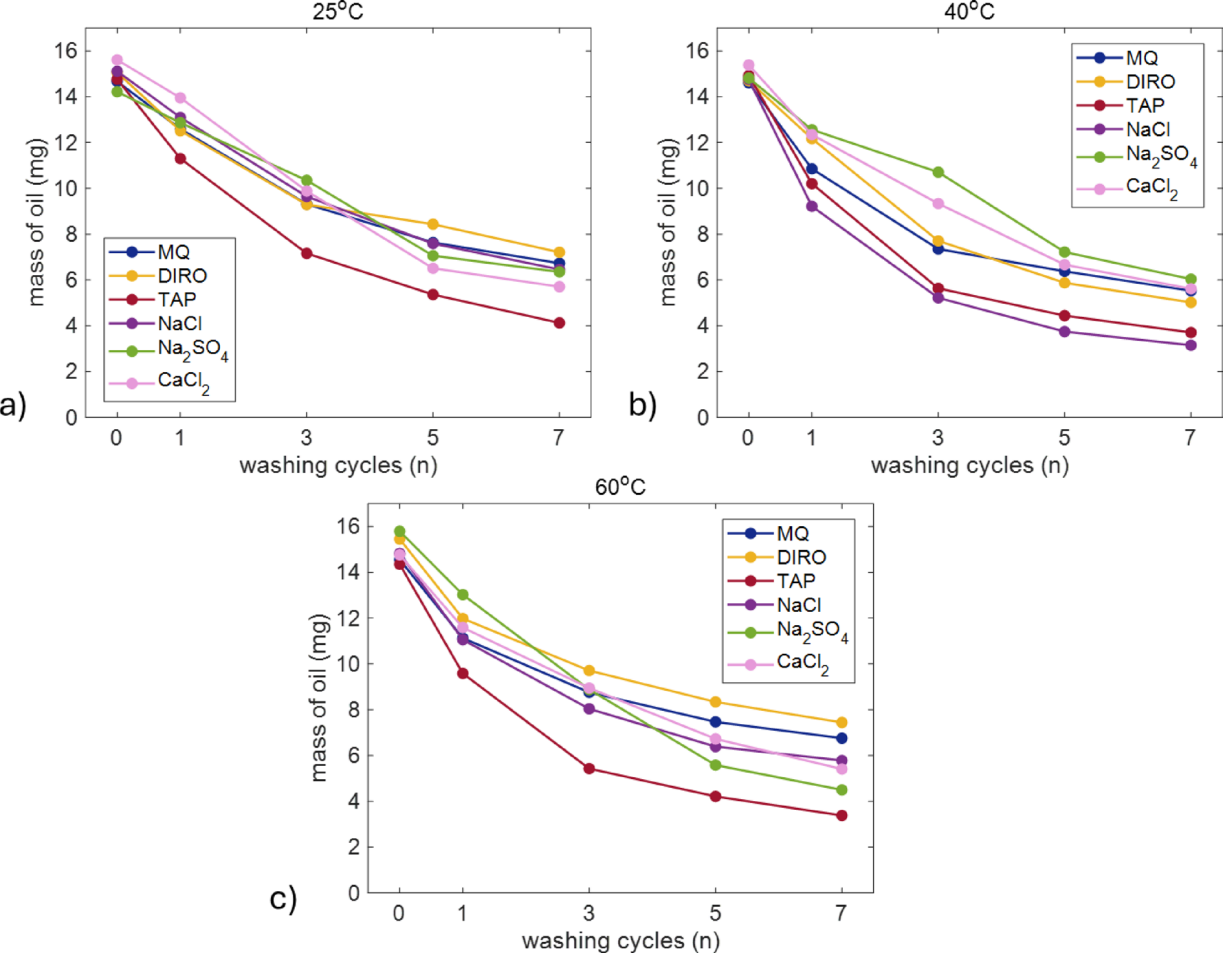
Average amount (mg) of oil left on the plastic surface after each washing cycle when different water grades (MQ, DIRO, TAP, NaCl, Na_2_SO_4_ and CaCl_2_ were used at **(a)** 25 °C **(b)** 40 °C **(c)** 60 °C. The number of replicates is 3 all apart from MQ and TAP (*n* = 6). Plots with error bars can be found in the supplementary data (Figure S3).

In the current system, since the absence of ions does not seem to be a primary factor facilitating oil removal, we hypothesized that the presence of ions, such as calcium and sodium, could influence TAP water’s cleaning efficiency. To test this, we conducted similar experiments using solutions containing 0.26 mmol of sodium sulphate (Na₂SO₄) and 0.25 mmol of calcium chloride (CaCl₂). These concentrations are close to those found in TAP water used in our experiments (Table S1). Figure 4 shows that the efficiency of these salt solutions does not match the one of TAP water, indicating that the presence of salt ions is not the primary reason for oil removal. To confirm that the concentration of ions is not the limiting factor, Na₂SO₄ solutions were also tested at three different concentrations: 0.10 mmol, 0.26 mmol, and 0.5 mmol. This variation in molarity allowed us to assess whether increasing the ion concentration would significantly impact the washing efficiency. However, as shown in Figure S4, the increase of ion concentration does not clearly influence oil removal.

### Washing solid surfaces with water at different pH values

Although the presence of ions did not cause a decrease of washing efficiency in case of hydrophobic surfaces, their presence can affect the process by influencing the pH of water. TAP and purified water grades often have different pH values due to their distinct compositions and treatment processes. In our case, the TAP water used in the experiments had a pH value of 7.9, making it slightly alkaline. This alkalinity is typically due to dissolved elements as indicated by the presence of calcium and magnesium ions, as well as the addition of alkaline substances during water treatment^23^. In contrast, purified water grades tend to have neutral (or slightly acidic) pH. This is primarily because the purification process, such as reverse osmosis or distillation, removes most minerals and dissolved solids from the water^24^. When the purified water leaves the purification system and comes in contact with air, the carbon dioxide can interact with the water and form carbonic acid. The carbonic acid can then dissociate in hydrogen and bicarbonate ions, which can make the pH of the purified grades slightly more acidic^25^.

#### Washing hydrophobic surfaces

To detect if this difference in the pH can affect the washing efficiency, different solutions were prepared by adding sodium hydroxide (NaOH) to water grades. The adjusted pH values ranged from 7.5 (for simplicity we refer them as pH 7) to 11 for different water grades (Table 1).

**Fig. 5:**
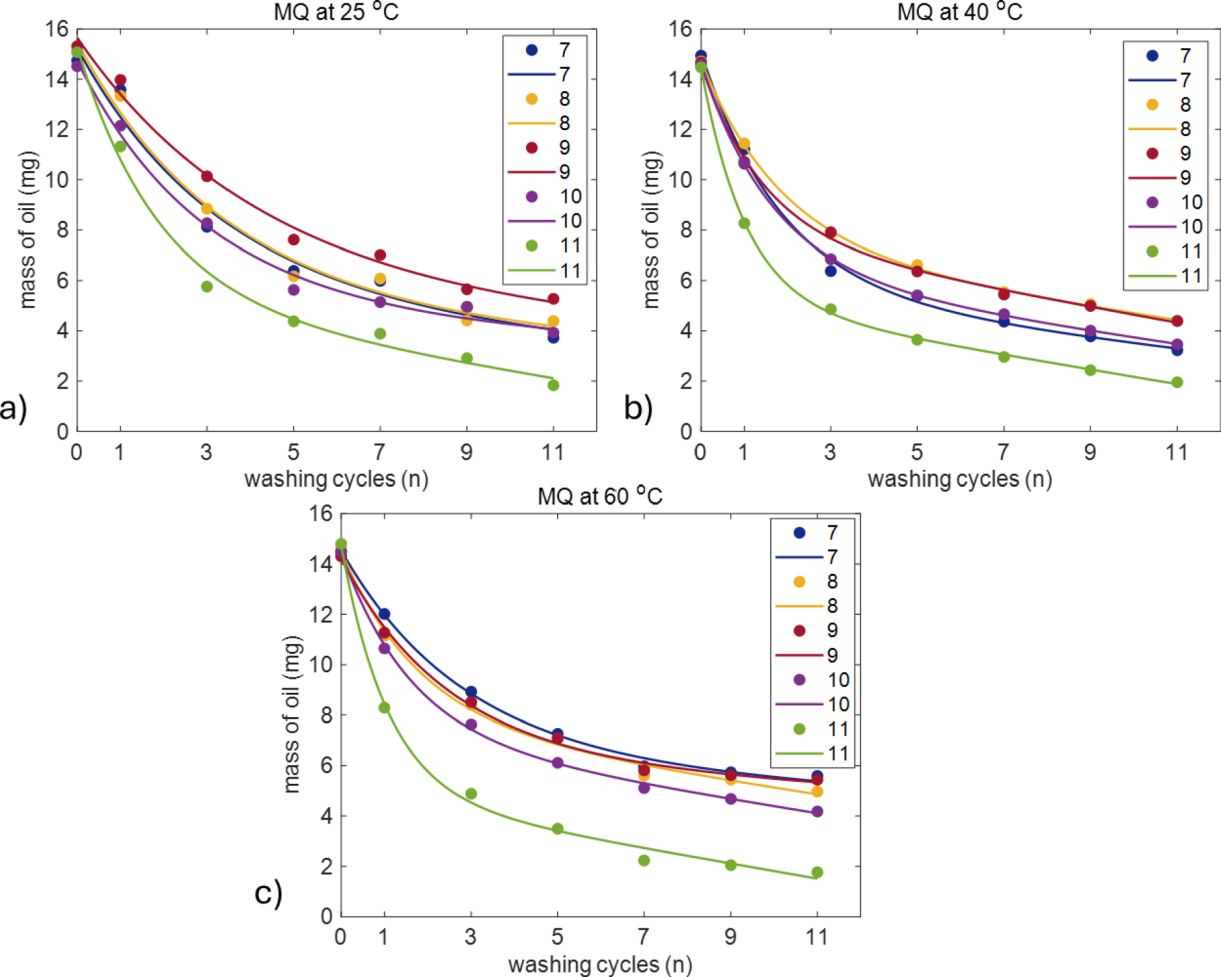
Comparison of washing with pH between 7–11 in MQ water at **(a)** 25 °C **(b)** 40 °C **(c)** 60 °C. Figures show the average amount (mg) of oil left on the plastic surface after each washing cycle. The number of replicates is 3. Plots with error bars can be found in the supplementary data (Figure S5). Data was fitted with Eq. (1).

For MQ water, the amount of residual oil on the plastic surface was measured after each washing cycle, at three different temperatures: 25 °C, 40 °C, and 60 °C (Fig. 5a-c). All plots exhibit a combination of an exponential and a linear decay, hence Eq. 1 was used to fit all data sets. The exponential regime is found between washing cycle 1 to 5 while after that, the linear regime follows.

Across all temperatures, the results show that higher pH solutions are significantly more effective at removing oil. Solutions with pH 11, which are more alkaline due to the presence of NaOH, removed oil more rapidly compared to less alkaline solutions such as the ones with pH 7 or 8. The solution with pH 11 (green curve) achieves the most rapid decrease in oil mass, with nearly all the oil being removed within 11 washing cycles. In contrast, the lower pH solutions, pH 7, 8 and 9 (blue, yellow and red curves), exhibit much slower oil removal, leaving almost twice as much oil on the surface under the same number of cycles compared to the higher pH solution.

**Fig. 6:**
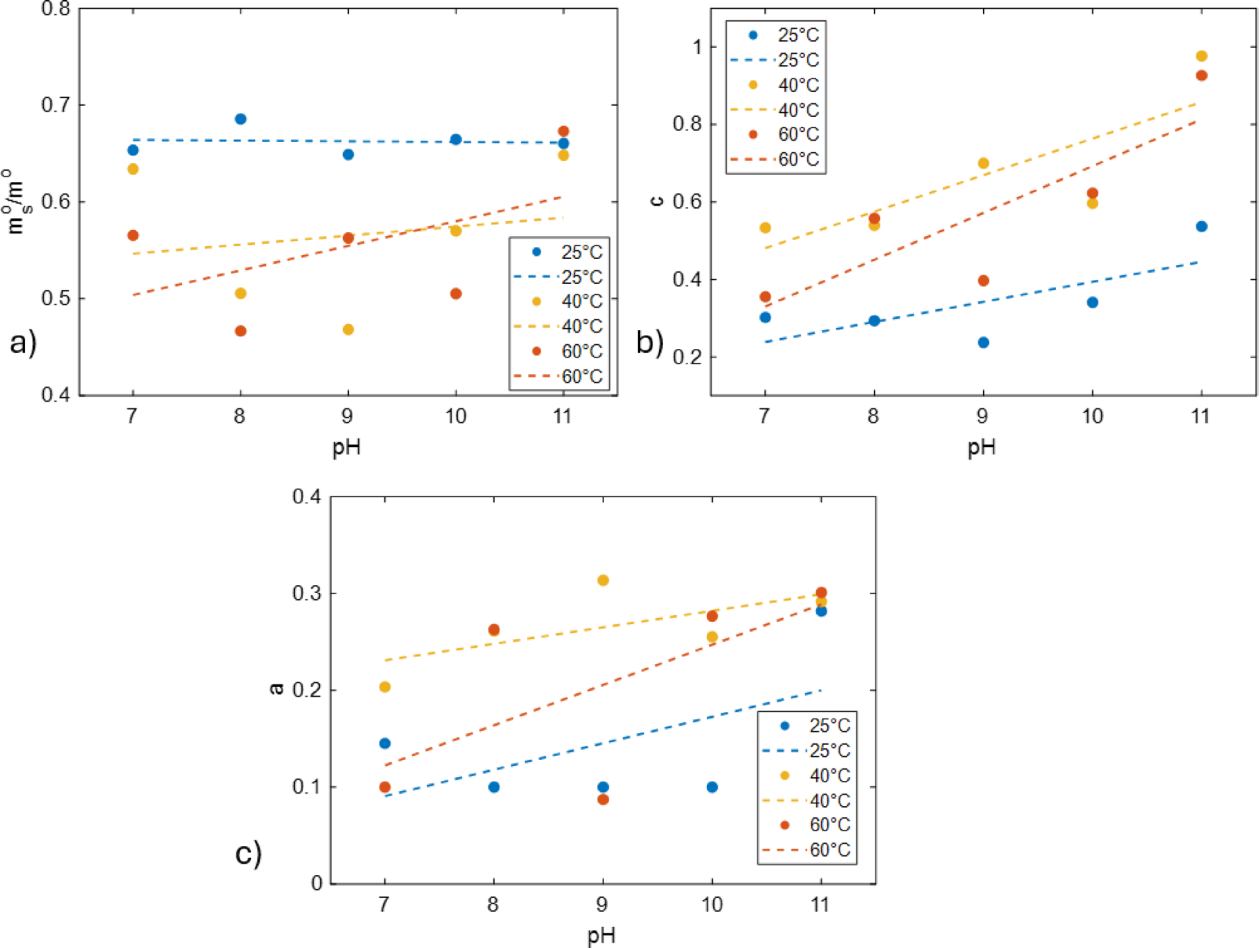
Parameters calculated from the non-linear fit of gravimetric data obtained from plastic tubes for MQ, at 25, 40 and 60 °C for pH 7 to 11; **(a**) mass of oil removed during the exponential regime **(b)** exponential decay **(c)** slope of the linear dependence. All parameters were obtained using Eq. (1). Dashed lines correspond to the linear regression fits for each temperature. Equations can be found in the supplementary data (Table S4).

To better understand how the pH affects the washing efficiency, the exponential and the linear terms obtained from the nonlinear fit were further analysed (Fig. 6). The fitted data shows the influence of pH on different regimes of the washing. The parameters of the exponential term are shown in Figs. 6a and b.

Both the mass of oil and the decay of the removal are affected by the pH (both between the same and different temperatures). At 25 °C, the mass of oil removed exhibits small variation with pH, which may be caused due to the limited number of washing cycles. At higher temperatures, pH promotes the removal of oil, with 60 °C having the steepest slope (Fig. 6a-b, Table S4), indicating the most pronounced difference in oil removal between lower and higher pH values. The second, linear, regime is affected as well by the pH. Regarding the fitting, the parameter *a* was given a limit value of 0.1. Due to limitations such as the number of washing cycles and the number of the data points, this parameter might not be as reliable as the others when it comes to 25 °C. Nonetheless, as seen from the fitting and the individual data points, pH promotes the removal of oil from the linear regime as well. As pH increased the amount of oil that is solubilised is increased, with 60 °C showing again the steepest slope (Table S4).

**Fig. 7:**
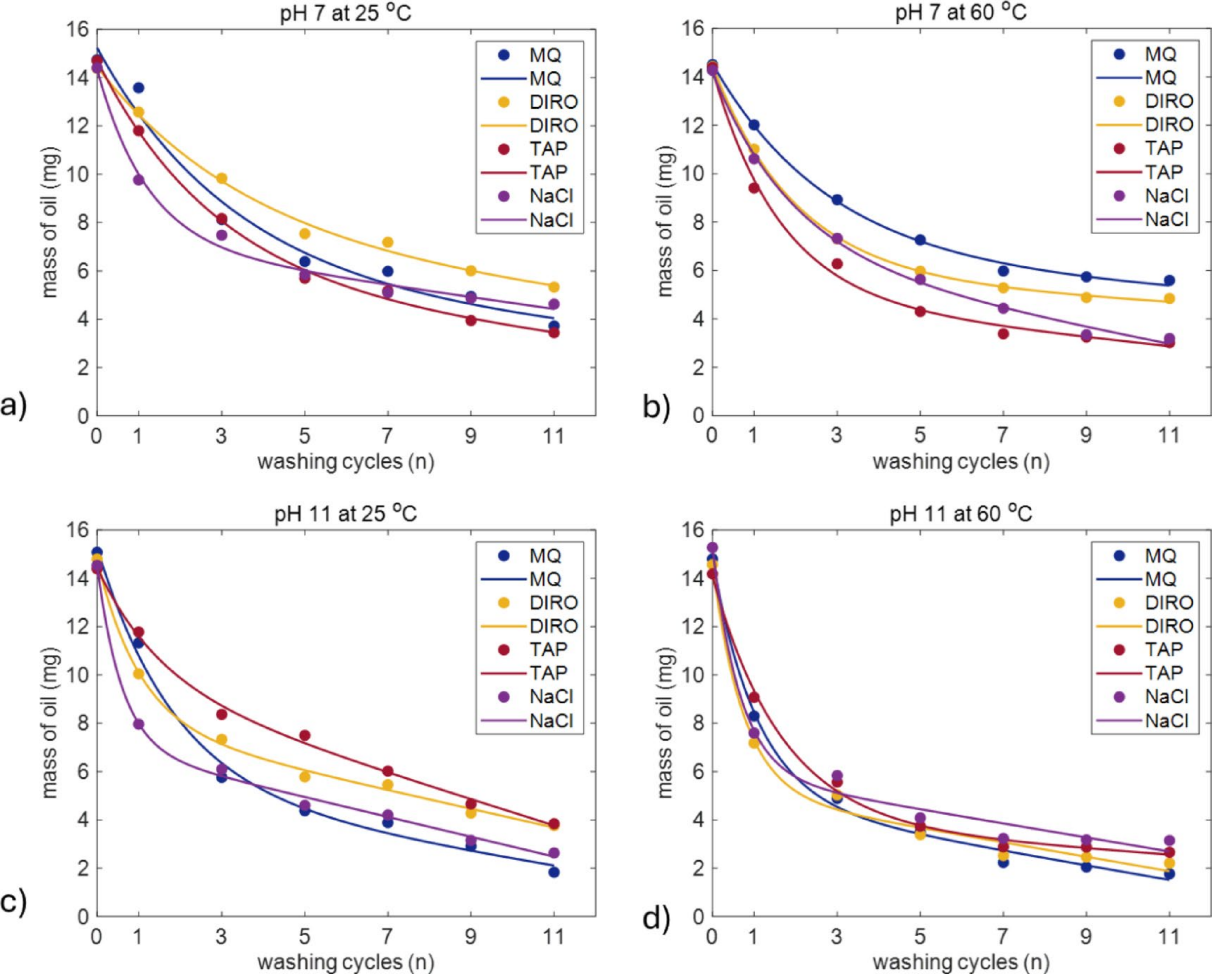
Comparison of washing with different water grades (MQ, DIRO, TAP, NaCl) with a) pH 7 and b)11 at 25 °C and with c) pH 7 and d)11 at 60 °C. Figures show the average amount of oil (mg) left on the plastic surface after each washing cycle. The number of replicates is 3. Plots with error bars can be found in the supplementary data (S8). Data was fitted with Eq. (1).

The efficiency of oil removal varies between different pH-adjusted water grades - MQ, DIRO, TAP, and NaCl solutions (Fig. 7), but less compared to variation in non-pH adjusted water grades (cf. Figure 1). This observation further illustrates the idea that pH is an important factor defining washing efficiency in this system - when variations in pH are eliminated, the difference between performances of water grades drops.

All water grades had their highest efficiency at high temperatures and pH 11(Fig. 7d, Figure S6). As it was discussed above using the MQ water example, these conditions facilitated both the surface (exponential regime) and bulk mechanisms (linear regime). However, it appears from comparison of Fig. 7b and c that the temperature and pH affect different water grades differently. In particular, increase of temperature makes TAP water most efficient while increase of pH makes MQ water most efficient. To understand the reason for this, it is instructive to recall the properties of water grades listed in Table 1. In the pH adjustment procedure, same amounts of NaOH were added to all water grades, which resulted in exactly the same pH at high alkalinity conditions (pH = 11.1 ± 0.05). For the case of more neutral conditions (denoted as pH 7 in Fig. 7) the pH variations were much higher due to stronger variations of the initial water grades properties. In particular, even after adjustment, pH of TAP water was highest, which explains why it performed best at in the data shown in Fig. 7b. In contrast, at pH 11 when much more NaOH was added, the original pH of water grades did not play a role and TAP water showed worst results (Fig. 7c), probably due to presence of divalent ions and other impurities. In Sect. “Surface tension mechanisms of oil removal under alkaline conditions” and “ Effect of alkalinity on washing mechanisms” we will further discuss how alkalinity affects the washing efficiency in this system.

Increasing the temperature from 25 °C to 60 °C, enhances the oil removal process. As mentioned before, at 25 °C (Fig. 5.a, Fig. 6), the initial step (exponential decay) of the washing process is slower across almost all pH levels, and solutions with higher pH, particularly pH 11, perform better. The data of MQ water showed that when the temperature increased to 40 °C, the oil removal rate increases, especially in more alkaline solutions (pH 9, 10 and 11). However, the differences between washing at 40 °C and at 60 °C are less pronounced. At these elevated temperatures, higher pH solutions (pH 10 and 11) exhibit nearly identical performance, while lower pH solutions (pH 7 to 9) show only minor improvements compared to their performance at 40 °C (Fig. 5). The temperature effect is more pronounced between 25 °C and 40 °C, whereas changes between 40 °C and 60 °C are less significant. In the studied temperature range, many properties of olive oil, such as viscosity, density, surface tension and oil–water interfacial tension tend to decrease as the temperature increases^26–29^.

An additional parameter that is affected, is the critical micelle concentration (CMC) of ionic amphiphiles present in our system. The CMC of ionic surfactants, including fatty acid salts, is temperature dependent and in most cases exhibits a U-shaped temperature dependence. With increased temperature the CMC decreases to a minimum, which can vary between 25 and 50 °C, then increases at higher temperatures^30–32^. The presence of a CMC minimum around 40 °C suggests that washing should be more effective at this temperature compared to 60 °C, where micelle formation is less favourable. However, other contributing factors such as reduced viscosity, lower interfacial tension and lower contact angle, facilitate oil removal upon heating. As a result, the overall washing efficiency strongly rises between 25 and 40 °C but does not show equal improvement upon further temperature increase to 60 °C.

The combined effect of high pH (pH 11) and increased temperature (60 °C) resulted in the most efficient oil removal across all water types. Under these conditions, all water grades almost completely removed the oil.

#### Washing hydrophilic surfaces

**Fig. 8:**
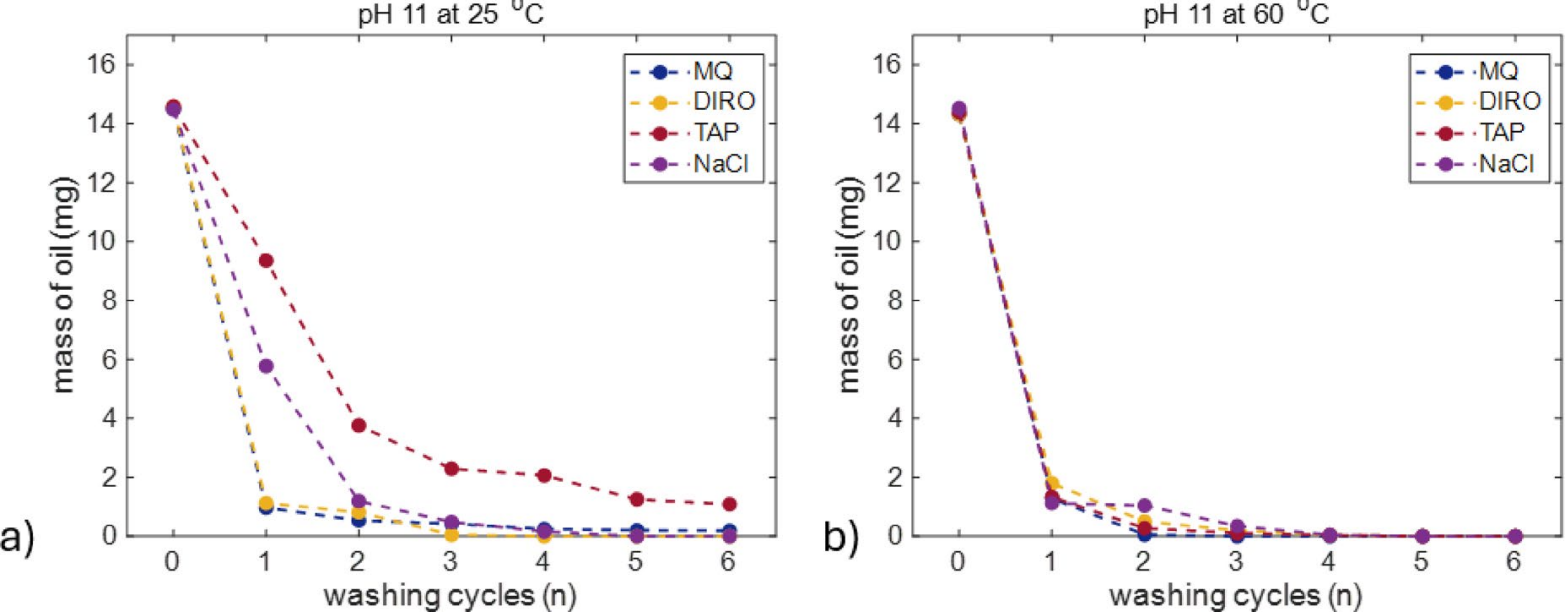
Average amount (mg) of oil left on the glass surface after each washing cycle with MQ, DIRO, TAP, NaCl water at pH 11 at **(a)** 25 °C **(b)** 60 °C. The number of replicates is 3. Plots with error bars can be found in the supplementary data (Figure S13).

Figure 8 shows the results on washing of different water grades when traces of NaOH were used to alter their pH.

At 25 °C (Fig. 8a) it is observed that the efficiency of the pH-adjusted purified water grades (MQ and DIRO) is higher than the efficiency of pH-adjusted TAP water. DIRO at pH 11, can completely remove the oil from the surface after 4 washing cycles and MQ water has an efficiency of 98.76% after 6 washing cycles. The pH adjusted NaCl solution completely removes everything after 5 cycles. Judging from the gravimetric experiments, pH-adjusted TAP water has the lowest efficiency. At 60 °C (Fig. 8b) all water grades exhibit maximum washing efficiency as after the 4th cycle all surfaces are completely clean.

**Fig. 9:**
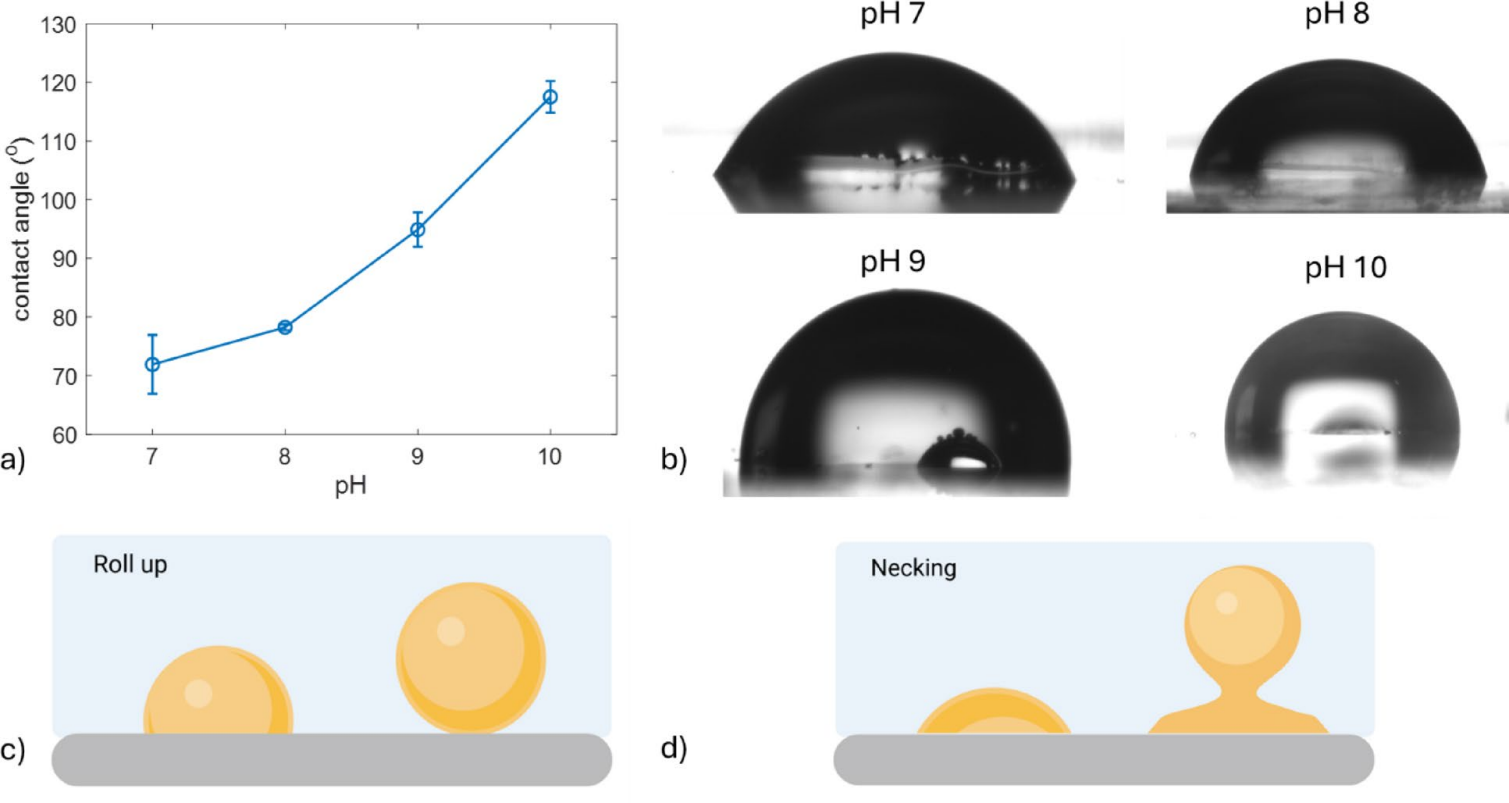
**(a)** Contact angle measurements of 5 µl olive oil droplet in MQ water with pH values of 7–10 (*n* = 3 for pH 7 and 8, *n* = 4 for pH 9 and 10). **(b)** Representative contact angle images of an olive oil droplet on a glass surface. Schematic representation of the: **(c)** roll up mechanism **(d)** necking mechanism of an oil droplet detachment from a surface in an aqueous media.

The data observed for pH 11 in both temperatures show that alkalinity is beneficial in washing hydrophilic surfaces. To get a better understanding of the oil removal process on a glass surface, the underwater contact angle of an olive oil droplet was measured at different pH values of MQ water. Figure 9 shows that as pH increased, the contact angle increased as well. When the pH of the water reached the value of 11, most of the volume of the droplet was quickly removed from the surface leaving behind a thin layer of oil. Due to the fast roll-up process, its parameters could not be monitored in this case.

#### Surface tension mechanisms of oil removal under alkaline conditions

In the topic of cleaning of solid surfaces, the roll-up mechanism is usually discussed as a key mechanism of oil removal. The roll up relies on the high contact angle of the oil in the three-phase system (surface, water, oil). High contact angle values can detach the oil either with or without the use of mechanical energy. Contact angles below 90^0^ are not sufficient to promote the detachment through this mechanism^33^. Still, the data presented here and in our previous works show that washing can occur in systems with contact angles below 90^0^.

**Fig. 10:**
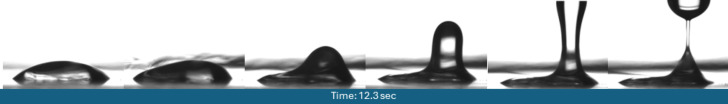
Removal of a 30 µl oil drop in MQ water when 30 µl of 1 M NaOH were added to the system. The NaOH was added drop by drop in the system from the top and gradually diffused downwards. The total duration of this experiment was 3 min during which the oil was removed through consecutive formation of four oil droplets.

To identify the mechanism of this process we performed an experiment where a drop of 30 µl of high concentration sodium hydroxide solution was added to water above oil drop (Fig. 10). The contact angle of the oil before addition of NaOH was 39.6^0^, and it remained constant or even decreased as the process proceeded. Nonetheless, when the NaOH reached the droplet, the interface started to deform, and oil was being removed from the surface still at low contact angle values. This suggests that another mechanism, and not the roll up, is responsible for the oil removal of this experiment.

As the NaOH approaches the droplet, the oil/water interfacial tension starts to change. The sodium hydroxide quickly deprotonates the free fatty acids and forms negative species which can adsorb at the oil-water interface and decrease locally the interfacial tension. According to Young’s equation^33^:

3 $$\:\text{c}\text{o}\text{s}{\uptheta\:}\:=\:\frac{\left({{\upgamma\:}}_{ws}\:-\:{{\upgamma\:}}_{os}\:\right)}{{\:{\upgamma\:}}_{\text{o}\text{w}\:}}\:\:\:\:\:\:\:\:\:\:\:$$

where *w* is water, *s* soil and *o* is olive oil, the contact angle is dependent on three interfacial tensions. In this experiment, however, deprotonation will quickly decrease the oil/water interfacial tension ($\:{\:{\upgamma\:}}_{\text{o}\text{w}\:}$in Eq. 3) due to it good accessibility, while the other two interfacial tensions will be affected later. This should increase the cos(θ) leading to the final decrease of the contact angle. Indeed, after the detachment of the first droplet (Figure S16), and when the system is more stable, the contact angle has decreased to 16^0^ (one should note that the difference between the equilibrium and receding contact angles can also have an effect). This confirms the idea that the roll up is not the mechanism responsible for the oil removal in this experiment.

The importance of this mechanism is found on the changes in the interfacial tension of oil in water ($\:{\:{\upgamma\:}}_{\text{o}\text{w}\:}$). As the deprotonated species reach the oil surface, the interfacial tension will decrease. To understand the system, we need to look at it through the Young–Laplace equation (Eq. 4).

4 $$\:\varDelta\:p=\rho\:gh-\gamma\:(\frac{1}{{R}_{1}}+\:\frac{1}{{R}_{2}})$$

Where $\:\varDelta\:p$ is the pressure difference between the two phases, *ρ* is the density difference, *g* is the gravity, *h* is the height, *γ* the interfacial tension and *R*_*1*_ and *R*_*2*_ are the radii. The two forces of importance are the interfacial tension and the gravity. If *γ* remains high, interfacial forces dominate and the droplet resists detachment. Reducing *γ* strengthens the relative effect of gravity, allowing oil (which has lower density than water) to detach forming free droplets. During the detachment, the oil is elongated, creates a ‘neck’ and then it pinches off. At certain stage the contact angle increases as well (53^0^, see Figure S16), indicating that more than one interfacial tension is affected.

To verify that this mechanism is indeed chemically driven, a control experiment was conducted, using a hexadecane droplet under identical conditions (Figure S17). In contrast to olive oil, the hexadecane droplet did not detach when NaOH was added, confirming that the deprotonation of fatty acids and the resulting decrease in interfacial tension are key factors in promoting droplet removal.

Overall, these observations illustrate that oil removal can be achieved without high contact angles values, but either gravity or a mechanical treatment involving acceleration (for example shaking) should be involved. To better understand how these mechanisms interact in practical washing systems where alkalinity is involved, we now shift to a broader discussion of all contributing processes.

#### Effect of alkalinity on washing mechanisms

With the mechanisms established, we now examine the broader impact of alkalinity on washing efficiency. Initial experiments of washing hydrophobic soils from hydrophobic surfaces showed that TAP water had a better efficiency, but after increasing the pH of purified water grades, this difference in the efficiency disappeared. Thus, the increased pH is the major factor improving efficiency in surfactant-free washing, which was clearly seen in case of both hydrophilic and hydrophobic surfaces. Below we will firstly describe two different chemical processes affected by pH – saponification and ionization. Then we discuss the surface-chemical (or colloidal) mechanisms influencing washing efficiency.

At higher pH more hydroxide ions are present, which facilitate hydrolysis of olive oil. Olive oil consists mainly of triglycerides (99%)^34^, which are made up of glycerol and fatty acids, free fatty acids, mono- and diacylglycerols, and a plethora of lipids such as hydrocarbons, sterols, aliphatic alcohols, tocopherols, and pigments^34^. In alkaline water, the hydroxide ions promote the saponification of the triglycerides, a process that involves the hydrolysis of the ester bonds within triglycerides (Eq. 5). In this reaction, the triglycerides break down and glycerol and fatty acid salts are formed. This process can occur in a stepwise manner, first producing diglycerides, followed by monoglycerides and finally glycerol. In all steps, the fatty acid salts are produced^35^:

5 $$\:{\left(RCOO\right)}_{3}{C}_{3}{H}_{5}+\:3{OH}^{-}\:\to\:\:3RCO{O}^{-}\:\left(soap\right)+\:{C}_{3}{H}_{5}{\left(OH\right)}_{3}\:\:\left(glycerol\right)$$

This chemical reaction requires rather high pH or longer reaction rates than the washing time used in this study. Therefore, since the effect of pH is seen already at much lower alkalinity, ester hydrolysis is probably not the only mechanism involved.

In addition to triglycerides, free fatty acids that are present in the oil, interact directly with hydroxide ions through deprotonation (Eq. 6). This process, leads to the formation of surface-active carboxylate ions (RCOO⁻) and water:

6 $$\:RCOOH\:\left(fatty\:acid\right)+O{H}^{-}\rightleftharpoons\:\:RCO{O}^{-}+\:{H}_{2}O$$

Both the saponification and the deprotonation result in the formation of ionised fatty acids. These anionic species consist of a hydrophobic hydrocarbon chain (fatty tail) and a hydrophilic head group (COO^−^)^18^. The negatively charged carboxylate group enables the fatty acid to interact with polar molecules, such as water, a property that is absent in neutral triglycerides and protonated free fatty acids due to their hydrophobicity. This characteristic makes the anionic fatty acids amphiphilic and surface active.

The pKa of oleic acid (~ 9.85)^36^ plays a crucial role in this process. Oleic acid, a major fatty acid in olive oil^34^, exists in its protonated form below its pKa, making it hydrophobic and poorly soluble in water. However, at pH 11, which is above its pKa, oleic acid fully deprotonates into its carboxylate form (oleate), increasing its solubility. The deprotonated form acts as a surfactant, which can facilitate both surface and bulk mechanisms of cleaning. Our data suggest that these deprotonated species increase washing efficiency in both the exponential and linear regime. For example, Fig. 6 shows that pH increase facilitates both the exponential (Fig. 6b) and linear (Fig. 6c) regimes, while the fraction that can be removed by surface mechanism (Fig. 6a) remains relatively constant.

The first mechanism that is affected by the pH and deprotonated fatty acids is related to contact angle. The contact angle of the oil increases with pH (Fig. 9) and extrapolation of this trend to pH 11 may produce a value close to 180 degrees, meaning the roll-up mechanism. At lower pH values and lower contact angles, the mechanical energy used in the experiments helps in removing the oil.

The second mechanism is related to the oil/water interfacial tension. Despite low contact angle values, the larger part of oil is removed from both hydrophobic and hydrophilic surfaces in the first (exponential) regime and addition of NaOH facilitates it. As shown in section “ Surface tension mechanisms of oil removal under alkaline conditions”., the deprotonated fatty acids can reduce the surface tension at oil-water interface by adsorbing at it, making it easier for the oil to increase the interfacial area. In other words, through the decrease in the surface tension which enables larger interfacial area between the liquids, oil droplets can be easier removed via the “necking” mechanism. 

The third mechanism could be related to interactions between interfaces close to the three-phase line between the solid, oil and water. The negatively charged surface active molecules bring negative charges to two interfaces: solid - water and oil – water. While the interactions between the charged interfaces would not act at distances visible in contact angle experiments, they can still play a role close to three phase line at smaller distances introducing additional repulsion between the droplet and the solid.

In the case of the hydrophilic surfaces, these mechanisms can fully remove the oil in a short number of cycles. In the case of the plastic surface that has a lower contact angle, the remaining oil could be removed through the solubilization process. The solubilization mechanism refers to the ability of oil molecules to be dissolved directly into the bulk or by being encapsulated in micelles^37,38^. Small amount of olive oil can be dissolved into the bulk, without the use of excess pH^14^. In the presence of surface-active molecules, such as deprotonated fatty acids, the solubilization of oil is facilitated by encapsulation in micelles.

While previous studies showed that washing thick layers of olive oil from hydrophobic surfaces is not effective without the use of surfactants^13^, these results indicate that alkalinity can increase the efficiency. Sodium hydroxide is a fully natural compound that consist of sodium ions present in large amounts in the environment (e.g. oceans) and hydroxide ions that are produced in self-ionisation of water. Unlike traditional washing with alkaline soap where substantial concentrations of surfactants are dissolved in bulk water, in the method described here they are produced almost exclusively at the oil-water interface – the place where they need to act. In this study we demonstrated an environmentally friendly washing using in situ production of eco-friendly surfactants in minimal quantities still sufficient for effective oil removal.

## Conclusions

In this study, we demonstrated how challenging it is to remove hydrophobic soils, like olive oil, from hydrophobic surfaces. To increase cleaning effectiveness in surfactant-free washing, several washing cycles and higher temperatures are needed. When removing the oil from the hydrophobic surface, tap water unexpectedly showed better efficiency than purified water grades. To find the reason for that, the influence of salts addition was investigated but no increase of efficiency was observed. Hence the effect was attributed to pH variation, and purified water’s cleaning effectiveness greatly increased when its pH was raised over 10.

We suggest that alkalinity deprotonates free fatty acids present in olive oil, producing surface active anions. Moreover, in the presence of hydroxide ions, triglycerides undergo saponification, breaking down into same carboxylate anions acting as natural surfactants. All these ionic species can remove the oil through surface mechanisms, such as the roll up and through surface tension changes. Increased temperatures further enhance this process by increasing the solubility of the soil, making it easier to wash it away. Increased alkalinity in the purified water grades not only improved the efficiency of washing hydrophobic surfaces but also achieved 100% cleaning efficiency on hydrophilic surfaces.

Although washing and cleaning with purified water grades is a relatively new process, in the last years we were able to show that their efficiency is sufficient to completely remove ‘hard’ stains such as olive oil from different surfaces. The mechanism of oil removal varies depending on the surface type but is now well understood. To advance this research, future studies should focus on the removal of oil from textiles, where different material properties may present additional challenges.

## Supplementary Information

Below is the link to the electronic supplementary material.

Supplementary Material 1

## Data Availability

The datasets used and/or analysed during the current study available from the corresponding author on reasonable request.
