# Supplementary material for: Optimizing mild surface cleaning methods: influence of water purity and pH
Source: Sci Rep. 2025 Aug 14;15:29815. doi: 10.1038/s41598-025-15143-0 (PMC12354923; doi:10.1038/s41598-025-15143-0)
Supplement: Supplementary file 1 — Supplementary Material 1 [file 41598_2025_15143_MOESM1_ESM.docx]

Supporting information

# Optimizing mild surface cleaning methods: Influence of water purity and pH

Andriani Tsompou^1,2^ and Vitaly Kocherbitov^1,2*^

^1^ Department of Biomedical Science, Malmö University, Malmö, Sweden

^2^ Biofilms research center for Biointerfaces, Malmö University, Malmö, Sweden

*Corresponding author. Department of Biomedical Science, Malmö University, SE-20506, Malmö, Sweden. Phone: +4640-6657946. E-mail: [Vitaly.Kocherbitov@mau.se](mailto:Vitaly.Kocherbitov@mau.se)

# Materials

**Table S1:** TAP water parameters taken from product report 2021 of Sydvatten for the water suppling the laboratory (Vombverket lake).

| Parameters | Unit | Median |
| --- | --- | --- |
| Temperature | ^o^C | 11 |
| Turbidity | FNU | < 0.10 |
| pH |  | 8.2 |
| Alkalinity (HCO^-^_3_) | mg/l | 140 |
| Hardness | ^o^dH | 6.5 |
| Calcium | mg/l | 36 |
| Magnesium | mg/l | 6.2 |
| Sodium | mg/l | 37 |

# Results

## Washing plastic tubes with different water grades


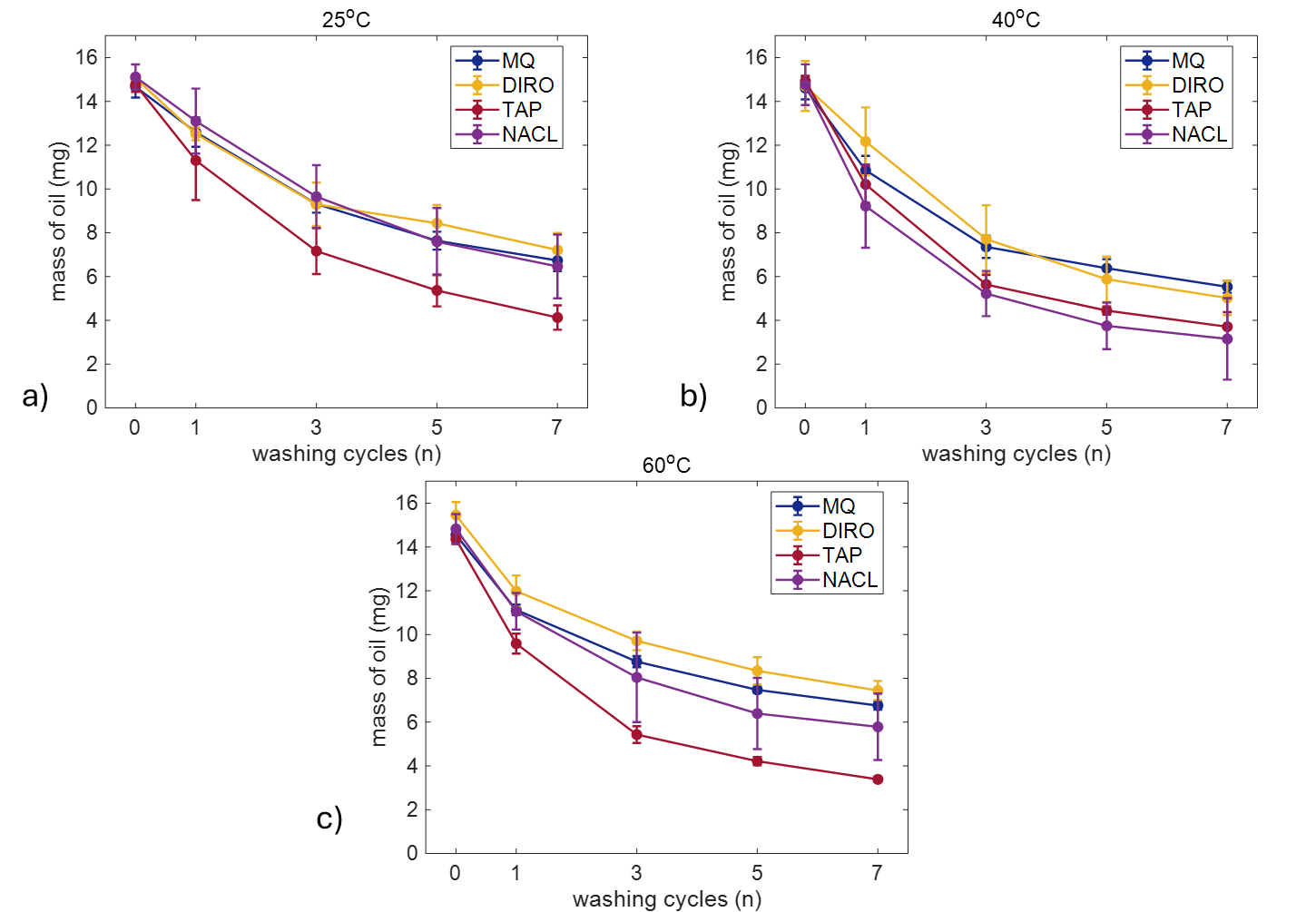


**Figure S1:** Average amount of olive oil left on the surface of plastic tubes (mg) as a function of the number of washing cycles for MQ, DIRO, NaCl, and TAP at a) 25 °C b) 40 °C and c) 60 °C with error bars. The number of replicates is 3 for DIRO and NaCl and 6 for MQ and TAP.


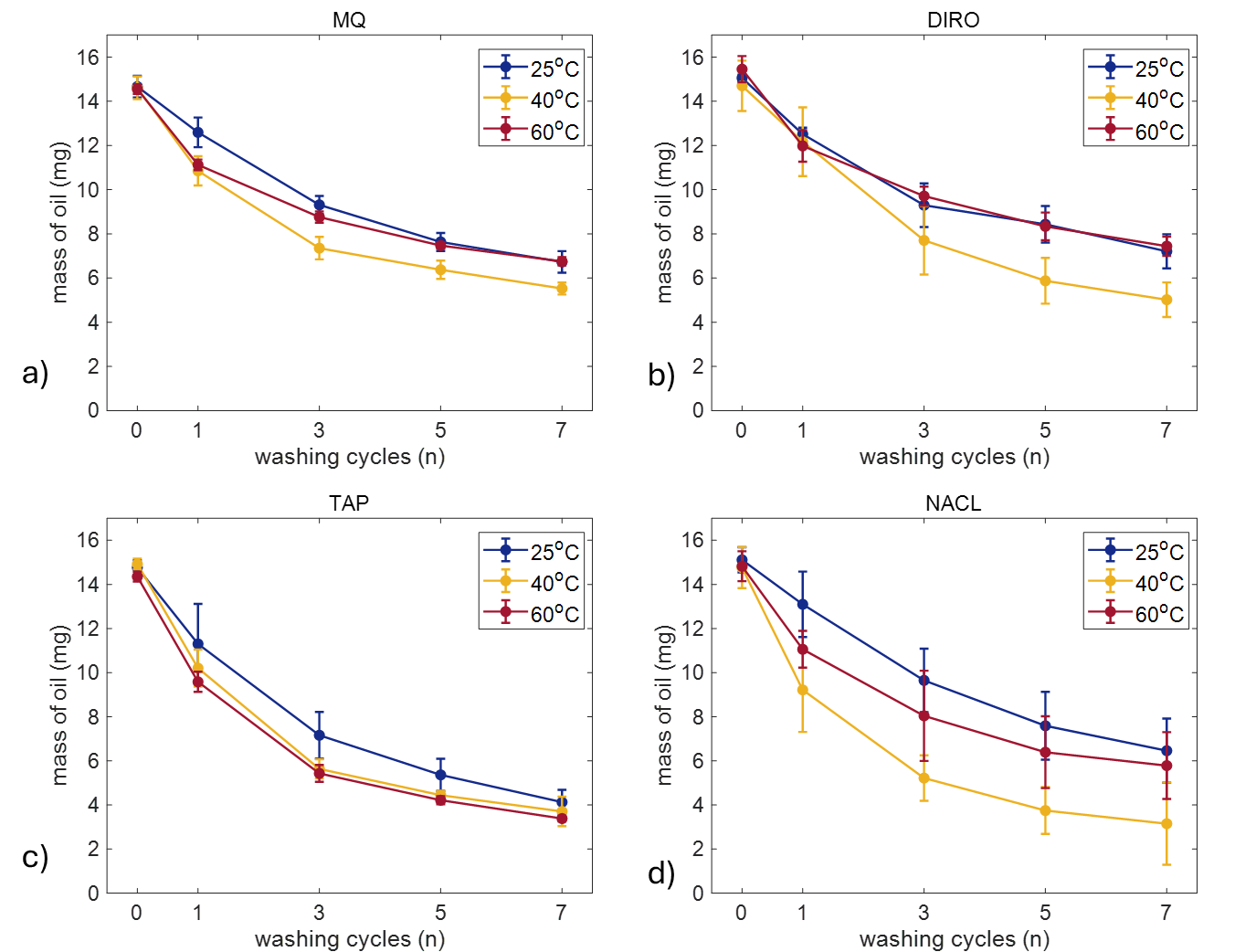


**Figure S2:** Average amount (mg) of oil left on the surface after each washing cycle when different water grades a) MQ, b) DIRO, c) TAP, d) NaCl were used at 25 °C, 40 °C, and at 60 °C. The number of replicates is 3 for DIRO and NaCl and 6 for MQ and TAP.

**Table S2:** Calculated values from non-linear fit of gravimetric data obtained from plastic tubes for MQ, DIRO, NaCl and TAP at 25, 40, and 60 °C. Only the exponential part of the equation is used.

| Temperature (°C) | Water grade | m^o^ | m^o^_s_ | c |
| --- | --- | --- | --- | --- |
| 25 | MQ | 14.7371 | 9.3676 | 0.2808 |
|  | DIRO | 15.0498 | 8.2688 | 0.3708 |
|  | TAP | 14.749 | 11.4992 | 0.3541 |
|  | NaCl | 15.1993 | 10.9915 | 0.2308 |
| 40 | MQ | 14.5803 | 9.1323 | 0.5153 |
|  | DIRO | 14.8716 | 11.1406 | 0.3228 |
|  | TAP | 14.9396 | 11.4097 | 0.5443 |
|  | NaCl | 14.6899 | 11.5442 | 0.6108 |
| 60 | M60 | 14.4251 | 7.7325 | 0.4878 |
|  | D60 | 15.2827 | 7.9211 | 0.4546 |
|  | T60 | 14.3208 | 11.0055 | 0.5511 |
|  | N60 | 14.7274 | 9.2185 | 0.4651 |

## Washing with divalent ions


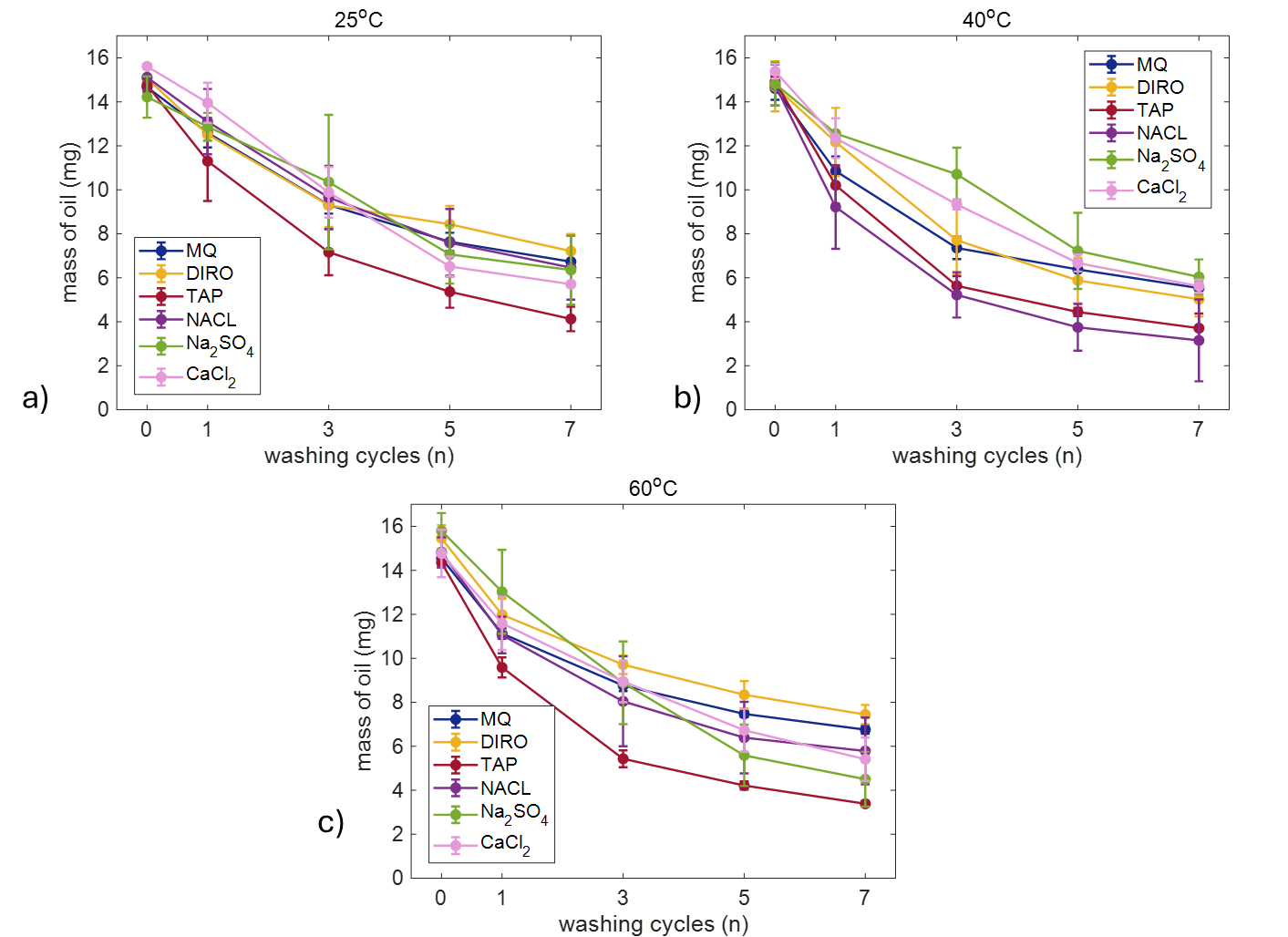


**Figure S3**: Average amount (mg) of oil left on the plastic surface after each washing cycle when different water grades (MQ, DIRO, TAP, NaCl, Na_2_SO_4_ and CaCl_2_ were used at a) 25 °C, b) 40 °C, and c) 60 °C. The number of replicates is 3 all apart from MQ and TAP (n = 6).


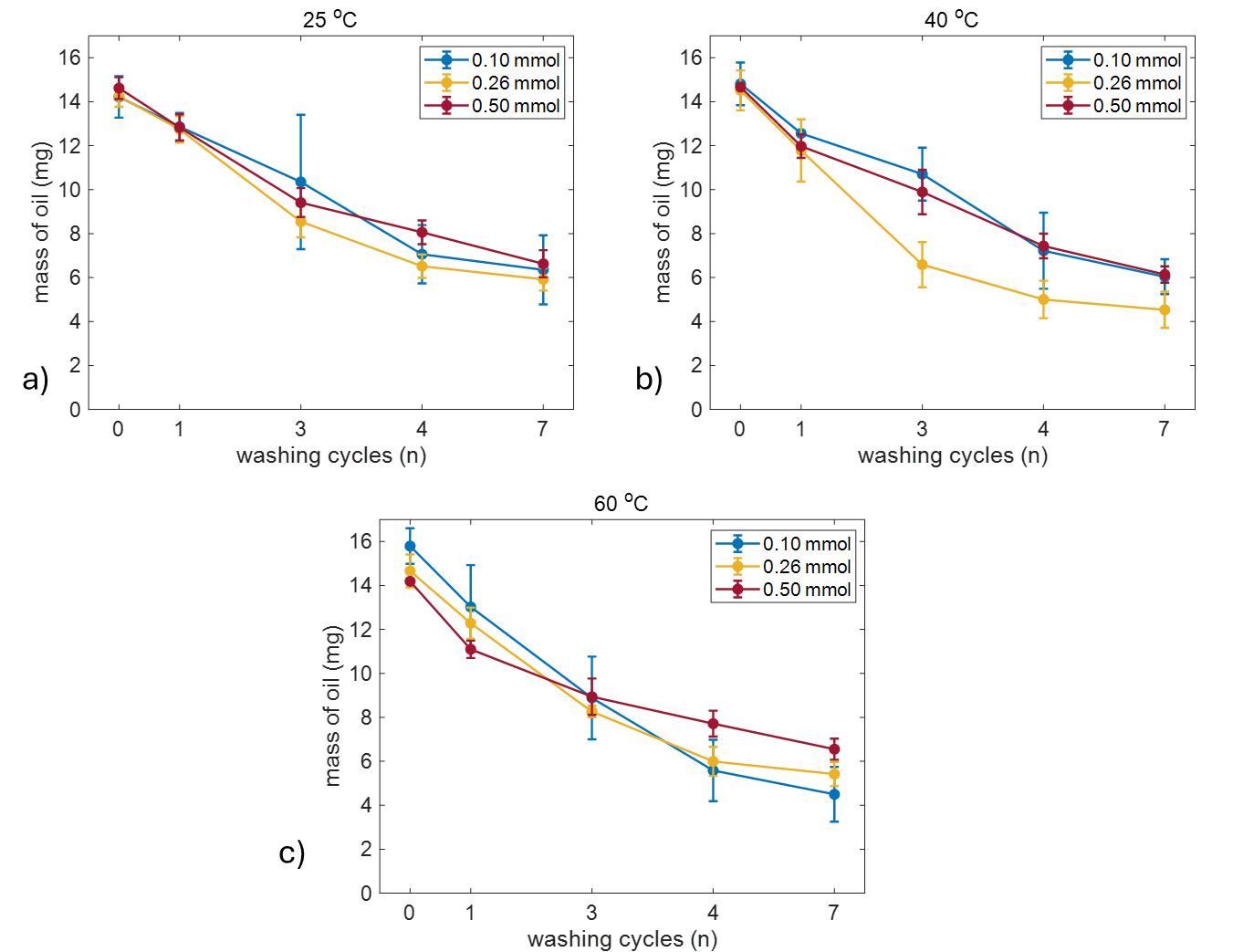


**Figure S4**: Average amount (mg) of oil left on the plastic surface after each washing cycle when Na_2_SO_4_ at 0.10, 0,26 and 0.50 mmol were used at a) 25 °C, b) 40 °C, and c) 60 °C.

## Washing with water grades at different pH levels

### Hydrophobic surfaces

#### 3.1.1 Different pH values in MQ water


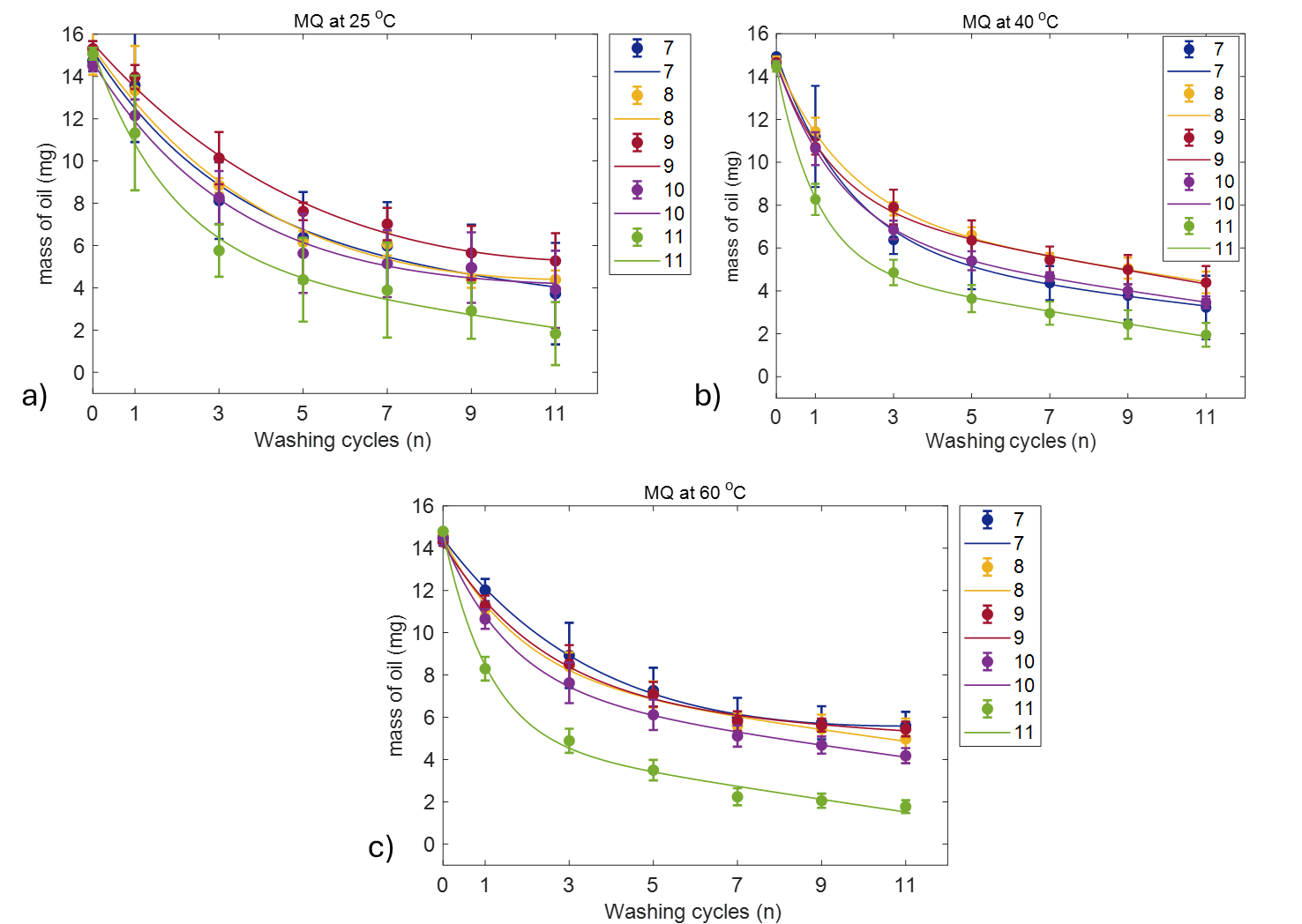


**Figure S5:** Average amount (mg) of oil left on the plastic surface after each washing cycle with MQ water at pH between 7-11 at a) 25 °C, b) 40 °C, and c) 60 °C. The number of replicates is 3.


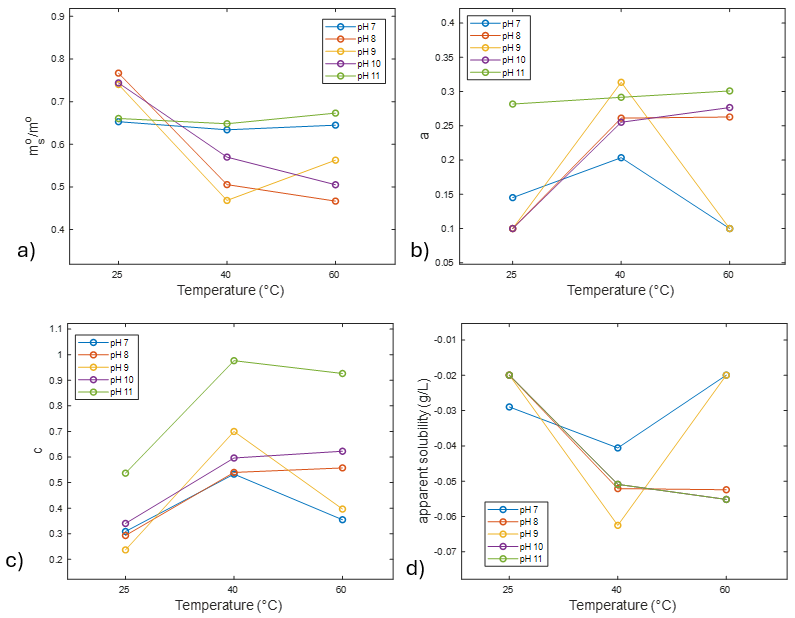


**Figure S6:** Parameters calculated from the exponential fit of gravimetric data obtained from plastic tubes for MQ at 25, 40 and 60 °C at pH 7-11; a) mass of oil removed during the exponential regime b) slope of the linear dependence c) exponential decay d) apparent solubility of olive oil obtained using equation (1).

**Table S3:** Calculated values from non-linear fit of gravimetric data obtained from plastic tubes for MQ, pH 7-11 at 25, 40, and 60 °C. $m^{o}$ is the initial mass of oil (mg), $m_{s}^{o}$ is the amount that can be removed during the exponential regime (mg), $c$ is the exponential decay constant, $a$ is the slope of the linear dependence.

| Temperature ( oC) | pH | mo | mos | c | a |
| --- | --- | --- | --- | --- | --- |
| 25 | 7 | 15.2343 | 9.9555 | 0.3091 | 0.1452 |
|  | 8 | 15.4696 | 10.6056 | 0.2934 | 0.1000 |
|  | 9 | 15.6373 | 10.1541 | 0.2371 | 0.1000 |
|  | 10 | 14.7212 | 9.7825 | 0.3405 | 0.1000 |
|  | 11 | 15.2714 | 10.0867 | 0.5369 | 0.2818 |
| 40 | 7 | 15.0470 | 9.5398 | 0.5332 | 0.2035 |
|  | 8 | 14.7341 | 7.4487 | 0.5399 | 0.2613 |
|  | 9 | 14.6172 | 6.8453 | 0.700 | 0.3136 |
|  | 10 | 14.5898 | 8.3167 | 0.5963 | 0.2553 |
|  | 11 | 14.4599 | 9.3729 | 0.9766 | 0.2917 |
| 60 | 7 | 14.5393 | 8.2215 | 0.3551 | 0.1000 |
|  | 8 | 14.5153 | 6.7750 | 0.5576 | 0.2629 |
|  | 9 | 14.2070 | 7.9939 | 0.3968 | 0.0873 |
|  | 10 | 14.4457 | 7.2986 | 0.6227 | 0.2767 |
|  | 11 | 14.7582 | 9.9333 | 0.9265 | 0.3010 |

**Figure S7:** Apparent solubility calculated from the non-linear fit of gravimetric data obtained from plastic tubes for MQ at 25, 40 and 60 °C for pH 7 to11 using equation (1).

**Table S4**: Linear regression equations from fitting of mass of oil removed during the exponential regime b) exponential decay c) slope of the linear dependence d) apparent solubilities (g/L) obtained using equation (1) data for MQ at 25, 40 and 60 °C.

| Data and temperature | Equation |
| --- | --- |
| m^o^_s_/m^o^ at 25°C | y = -0.0007x + 0.6690 |
| m^o^_s_/m^o^ at 40°C | y = 0.0093x + 0.4816 |
| m^o^_s_/m^o^ at 60°C | y = 0.0254x + 0.3263 |
| c at 25°C | y = 0.0517x - 0.1236 |
| c at 40°C | y = 0.0943x - 0.1797 |
| c at 60°C | y = 0.1208x - 0.5154 |
| a at 25°C | y = 0.0273x - 0.1005 |
| a at 40°C | y = 0.0170x + 0.1117 |
| a at 60°C | y = 0.0416x - 0.1686 |
| Apparent solubility (g/L) at 25°C | y = 0.0054x - 0.0200 |
| Apparent solubility (g/L) at 40°C | y = 0.0034x + 0.0223 |
| Apparent solubility (g/L) at 60°C | y = 0.0083x - 0.0336 |

#### 3.1.2 Different water grades in pH 7 and 11


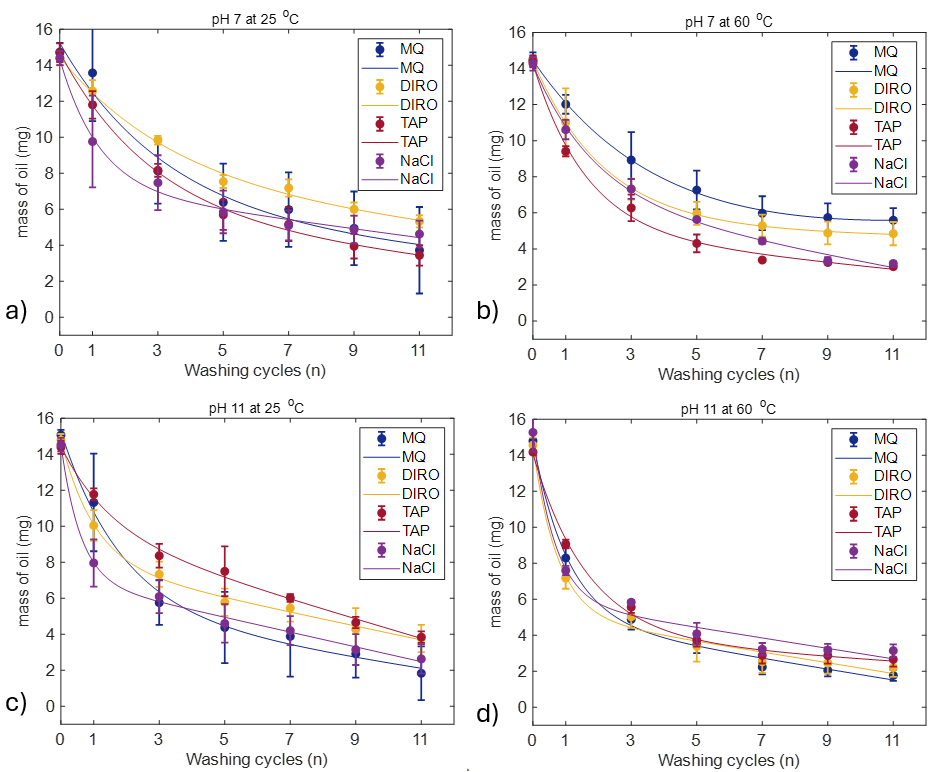


**Figure S8:** Average amount (mg) of oil left on the plastic surface after each washing cycle with MQ, DIRO, TAP, NaCl water at pH between 7 and 11 at a,c) 25 °C, b,d) 60 °C. The number of replicates is 3. Data was fitted with equation (1).

**Table S5:** Calculated values from non-linear fit of gravimetric data obtained from plastic tubes for DIRO, TAP, NaCl, pH 7 and 11 at 25, and 60 °C. $m^{o}$ is the initial mass of oil (mg), $m_{s}^{o}$ is the amount that can be removed during the exponential regime (mg), $c$ is the exponential decay constant, $a$ is the slope of the linear dependence.

| Water grade | pH | Temperature ( oC) | mos/mo | c | a |
| --- | --- | --- | --- | --- | --- |
| DIRO | 7 | 25 | 0.5131 | 0.2773 | 0.1814 |
|  | 7 | 60 | 0.6445 | 0.4578 | 0.035 |
|  | 11 | 25 | 0.4625 | 0.9864 | 0.3873 |
|  | 11 | 60 | 0.6456 | 1.349 | 0.298 |
| TAP | 7 | 25 | 0.635 | 0.3504 | 0.1923 |
|  | 7 | 60 | 0.6598 | 0.6187 | 0.1814 |
|  | 11 | 25 | 0.328 | 0.6668 | 0.54 |
|  | 11 | 60 | 0.7261 | 0.6008 | 0.1187 |
| NaCl | 7 | 25 | 0.5026 | 0.8373 | 0.2463 |
|  | 7 | 60 | 0.5331 | 0.5237 | 0.335 |
|  | 11 | 25 | 0.5184 | 1.681 | 0.4108 |
|  | 11 | 60 | 0.614 | 1.4963 | 0.291 |


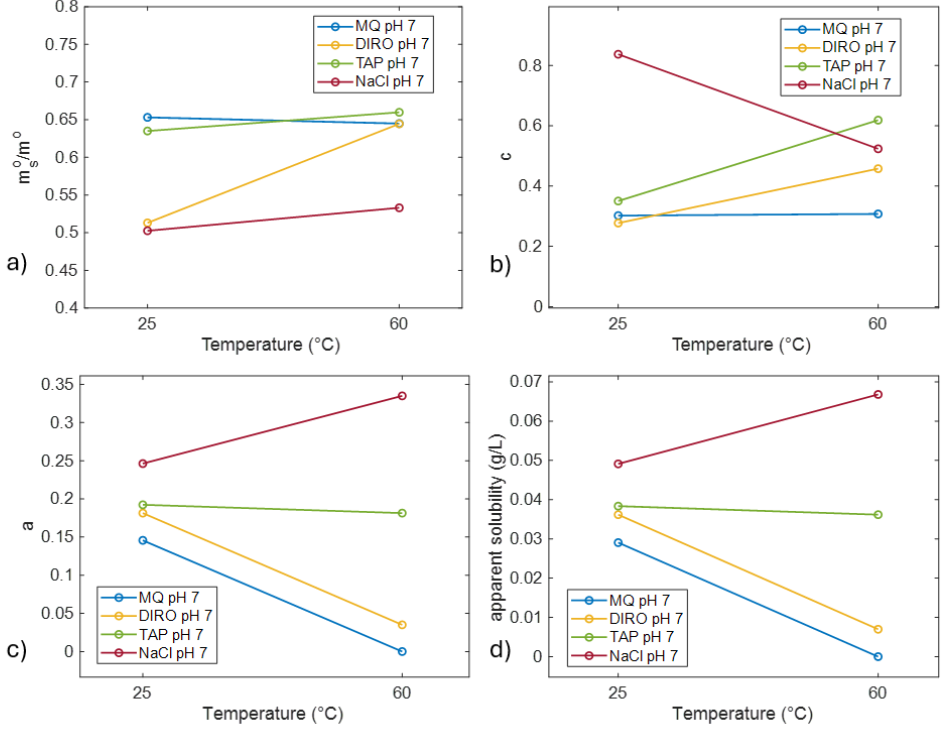


**Figure S9:** Parameters calculated from the non-linear fit of gravimetric data obtained from plastic tubes for MQ, DIRO, NaCl and TAP at 25 and 60 °C at pH 7; a) mass of oil removed during the exponential regime b) exponential decay c) slope of the linear dependence d) apparent solubilities (g/L) obtained using equation (1).


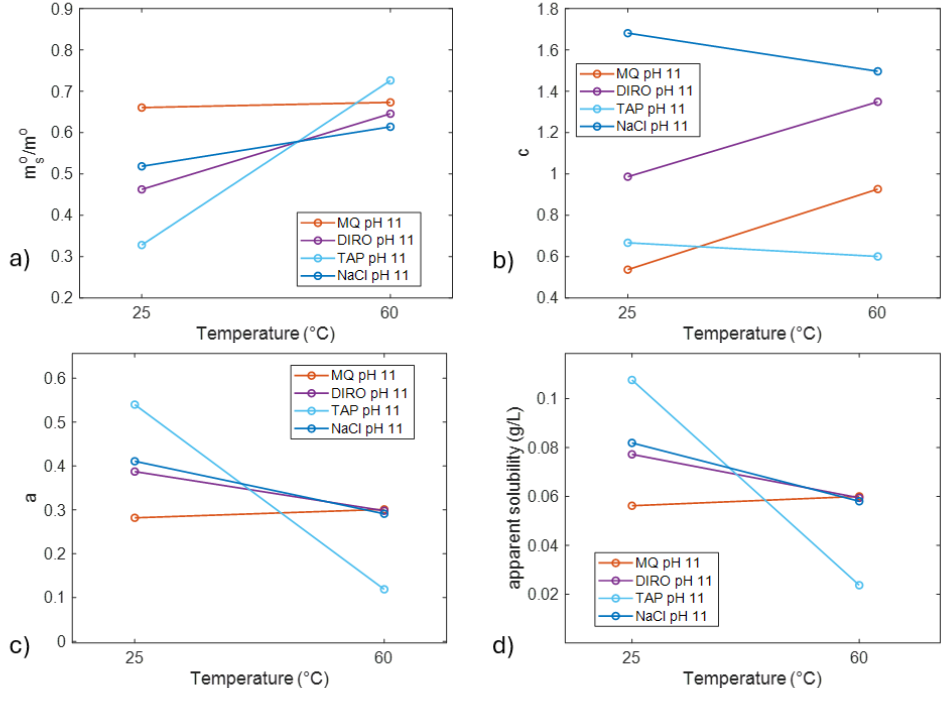


**Figure S10:** Parameters calculated from the non-linear fit of gravimetric data obtained from plastic tubes for MQ, DIRO, NaCl and TAP at 25 and 60 °C at pH 11; a) mass of oil removed during the exponential regime b) exponential decay c) slope of the linear dependence d) apparent solubilities (g/L) obtained using equation (1).


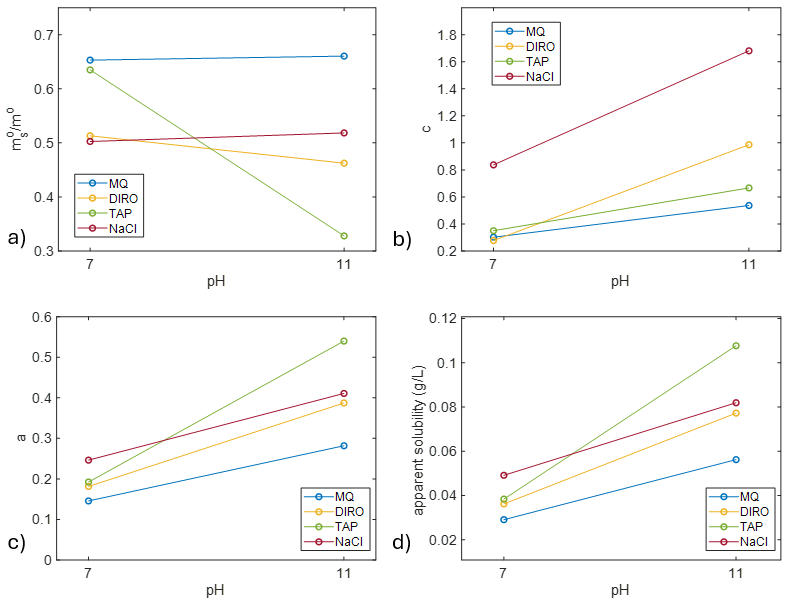


**Figure S11:** Parameters calculated from the non-linear fit of gravimetric data obtained from plastic tubes for MQ, DIRO, NaCl and TAP at 25 °C for pH 7 and 11; a) mass of oil removed during the exponential regime b) exponential decay c) slope of the linear dependence d) apparent solubilities (g/L) obtained using equation (1).


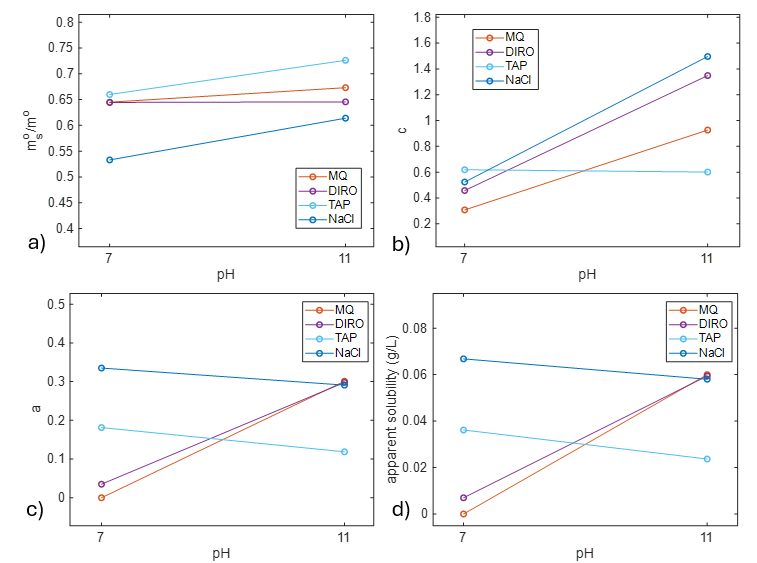


**Figures12:** Parameters calculated from the non-linear fit of gravimetric data obtained from plastic tubes for MQ, DIRO, NaCl and TAP at 60 °C for pH 7 and 11; a) mass of oil removed during the exponential regime b) exponential decay c) slope of the linear dependence d) apparent solubilities (g/L) obtained using equation (1).

### 3.2 Hydrophilic surface


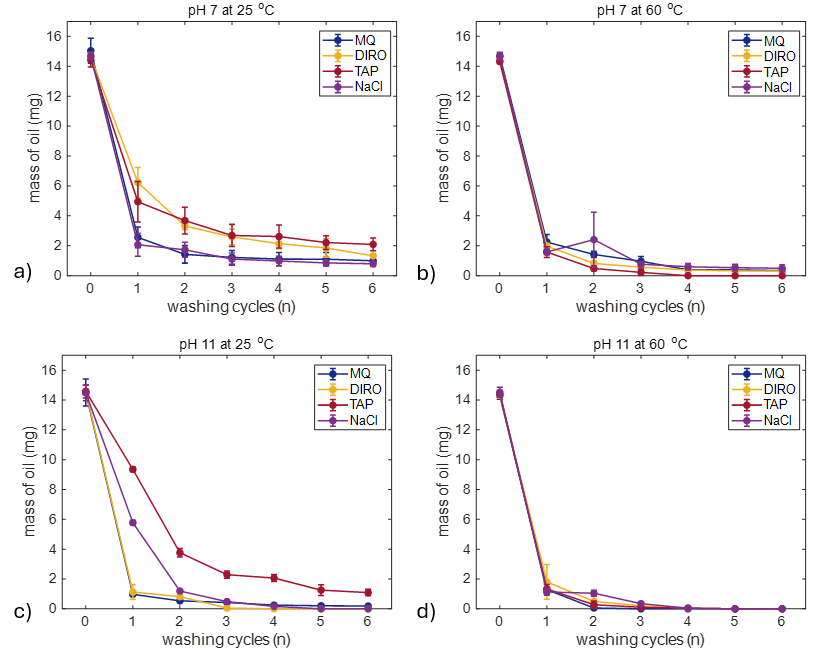


**Figure S13:** Average amount (mg) of oil left on the glass surface after each washing cycle with MQ, DIRO, TAP, NaCl water at pH 7 at a) 25 °C, b) 60 °C and at pH 11 at d) 25 °C, d) 60 °C. The number of replicates is 3.


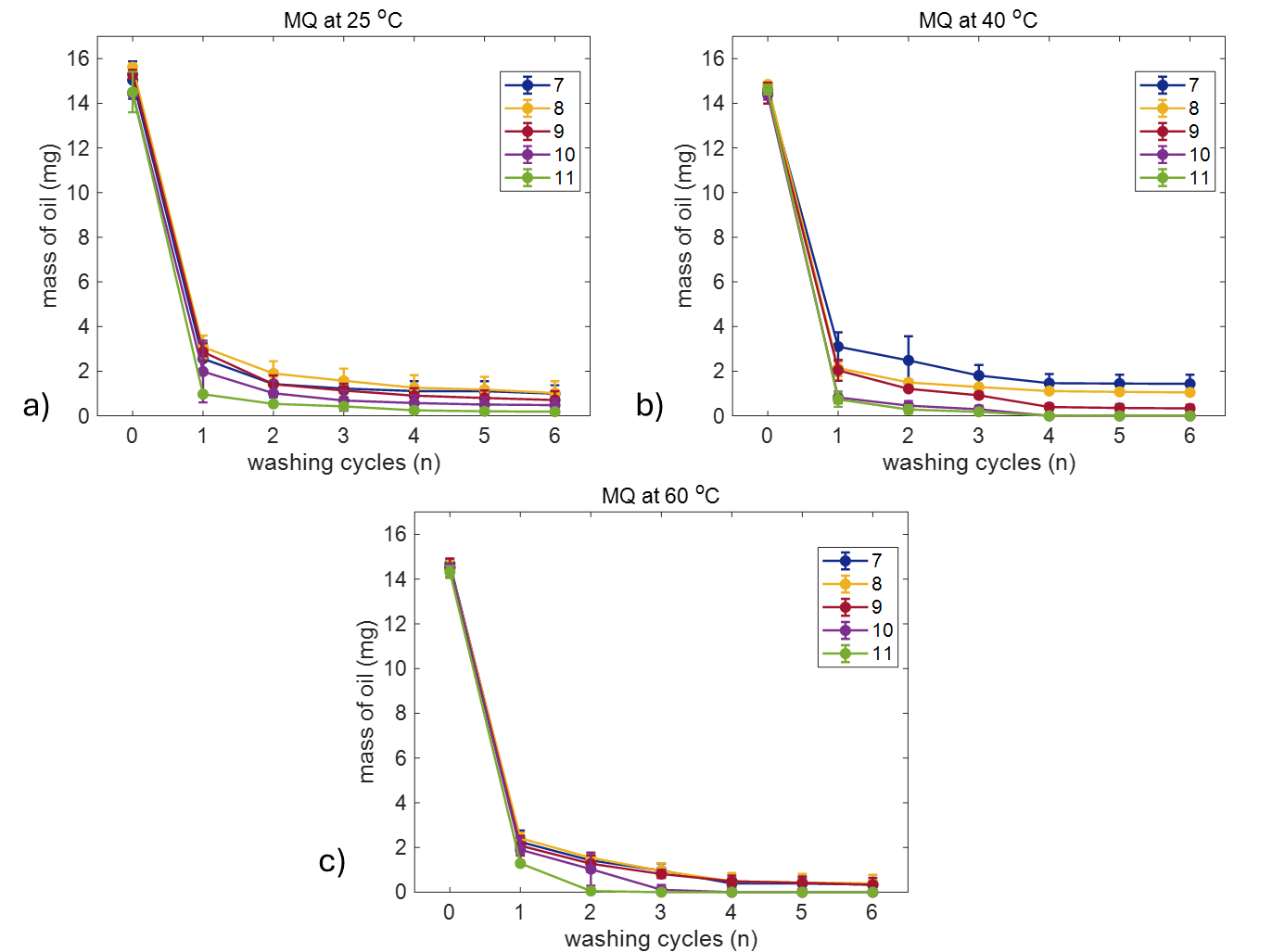


**Figure S14:** Average amount (mg) of oil left on a glass surface after each washing cycle with MQ water at pH between 7-11 at a) 25 °C, b) 40 °C, and c) 60 °C. The number of replicates is 3. Plots with error bars can be found in the supplementary data.

## Characterization of plastic tubes


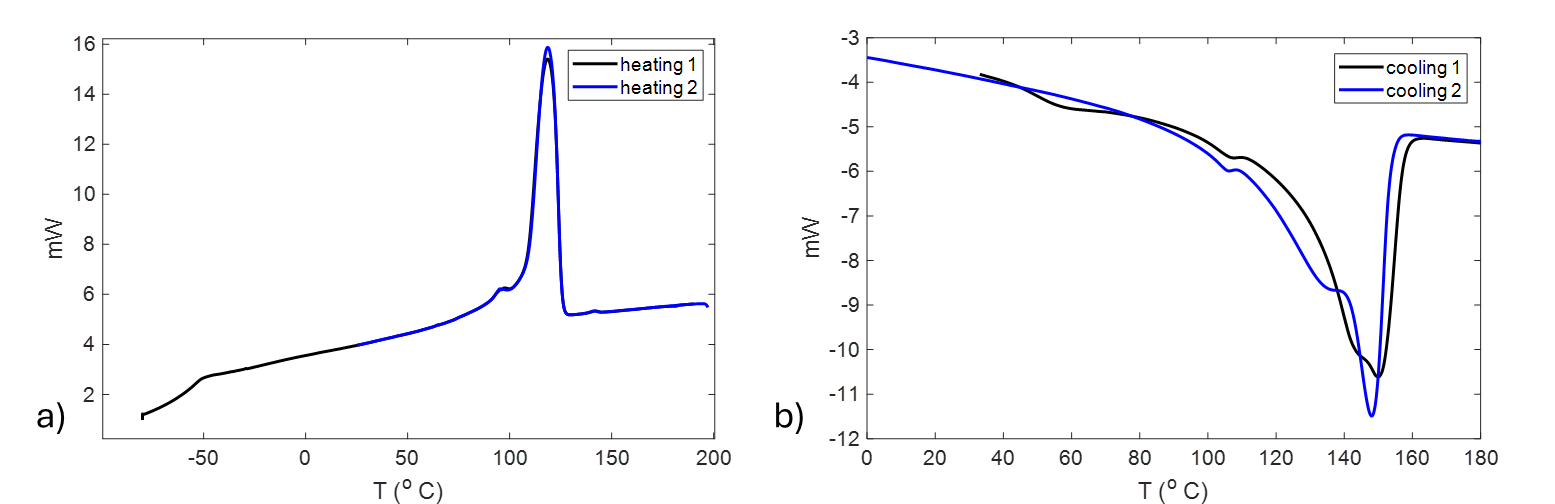


**Figure S15:** Differential Scanning Calorimetry (DSC) curve of plastic tubes during heating (a) and cooling cycles (b).

## Surface tension mechanisms of oil removal under alkaline conditions


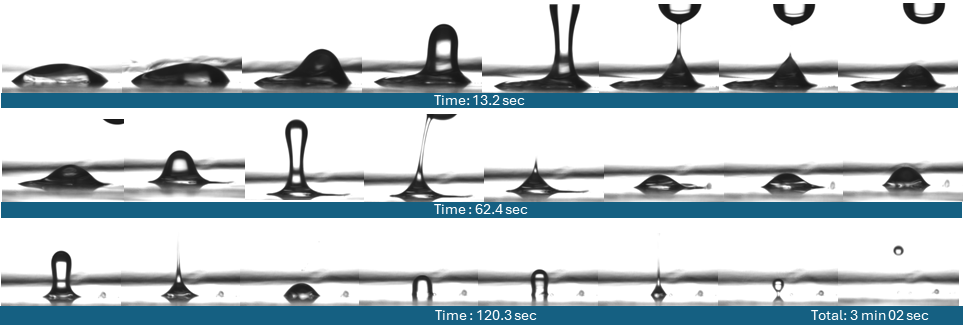
**Figure S16:** Removal of a 30 μl oil drop in MQ water when 30 μl of 1M NaOH were added to the system. The NaOH was added drop by drop in the system from the top. The total duration of the mechanism is 3 minutes during which the oil was removed through the formation of four separate oil droplets.


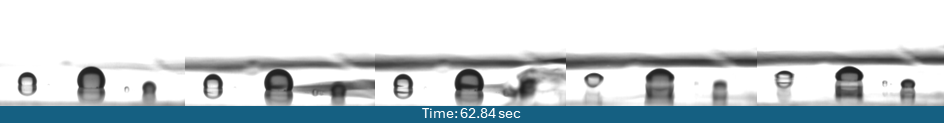


**Figure S17:** Removal of a 40 μl hexadecane drop in MQ water when 30 μl of 1M NaOH were added to the system. The NaOH was added drop by drop in the system from the top. The total duration of the mechanism is 62 seconds during which the hexadecane was not removed from the surface.

## AFM on glass slides


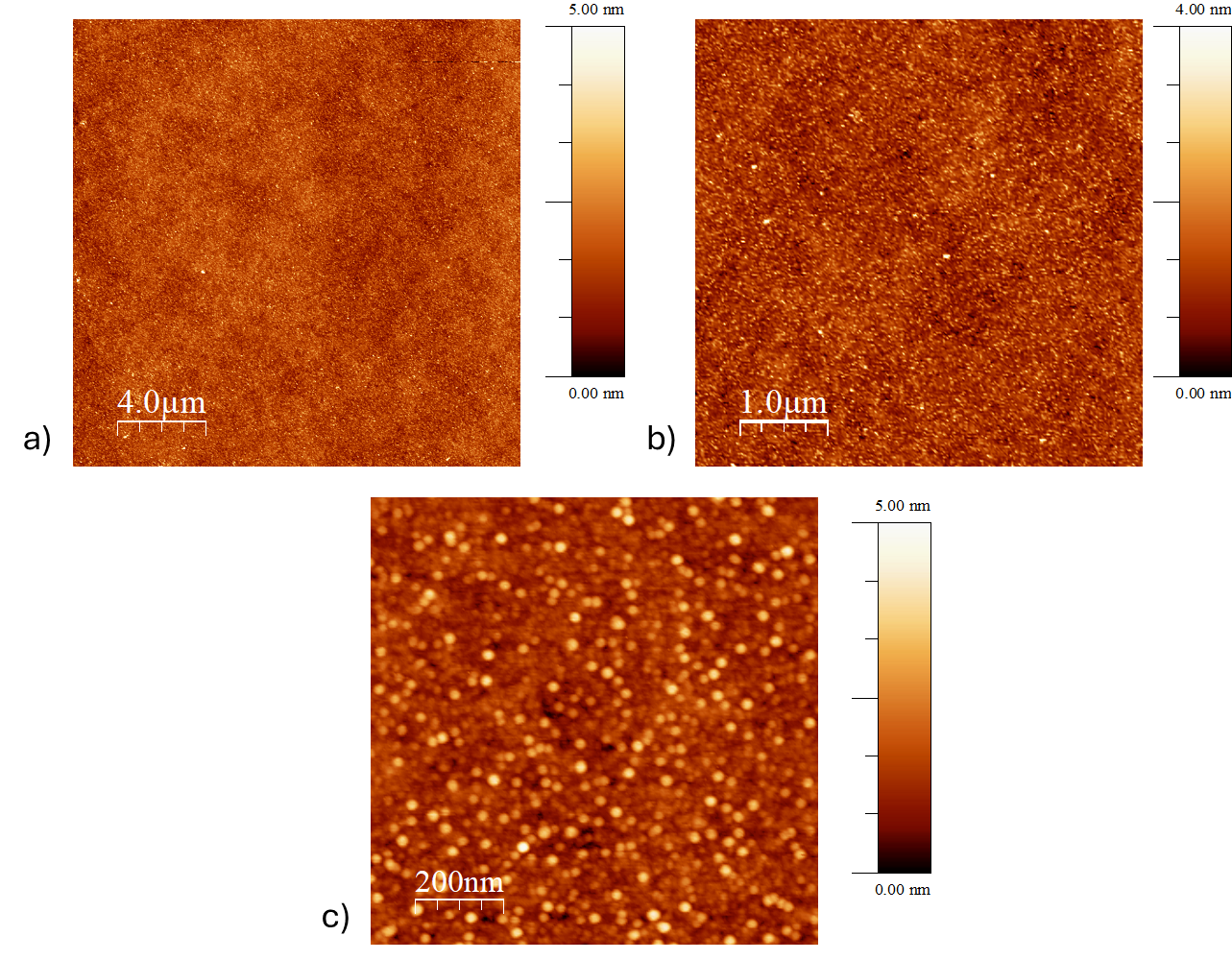


Figure S18: AFM topographic characterization of glass substrates. Tapping mode was used. 3 different magnifications were tested; a) 4.0 μm b) 1 μm c) 200 nm scale bars.

In figure S18a the region that was studied was 20 μm x 20 μm and a total of 512 x 512 points were taken. The average height was 1.65 nm and the average roughness 0.50 nm. For figure S18b, the region that was studied was 5 μm x 5 μm and a total of 256 x 256 points were taken. The average height was 1.13 nm and the average roughness 0.50 nm. . For figure S18c, the region that was studied was 1 μm x 1 μm and a total of 256 x 256 points were taken. The average height was 1.40 nm and the average roughness 0.60 nm. All respective histograms can be found in figures S19-S21.


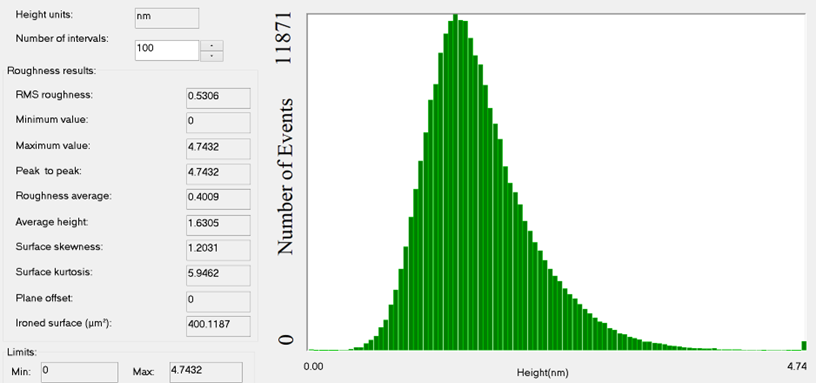


Figure S19: AFM histogram corresponding to sample of figure S19a.


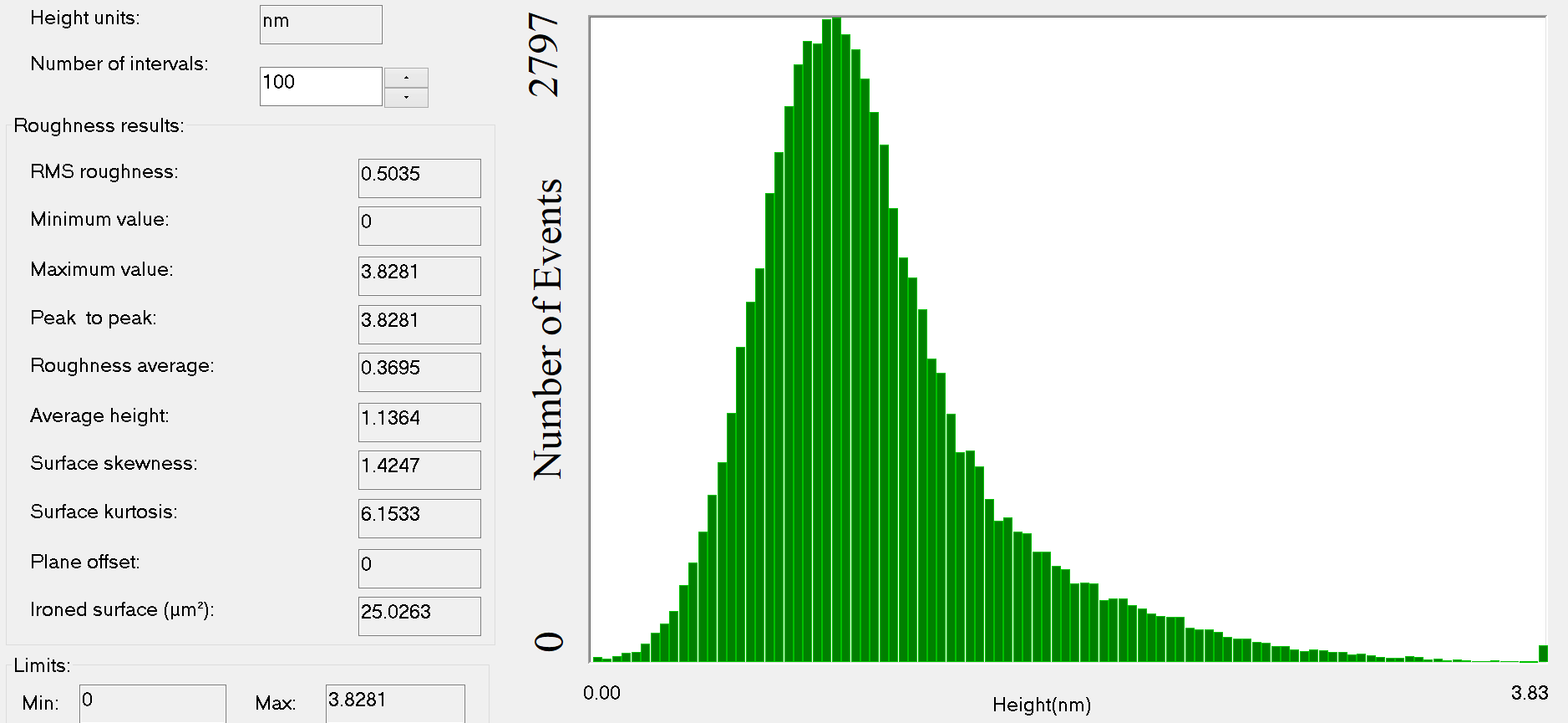


Figure S20: AFM histogram corresponding to sample of figure S19b.


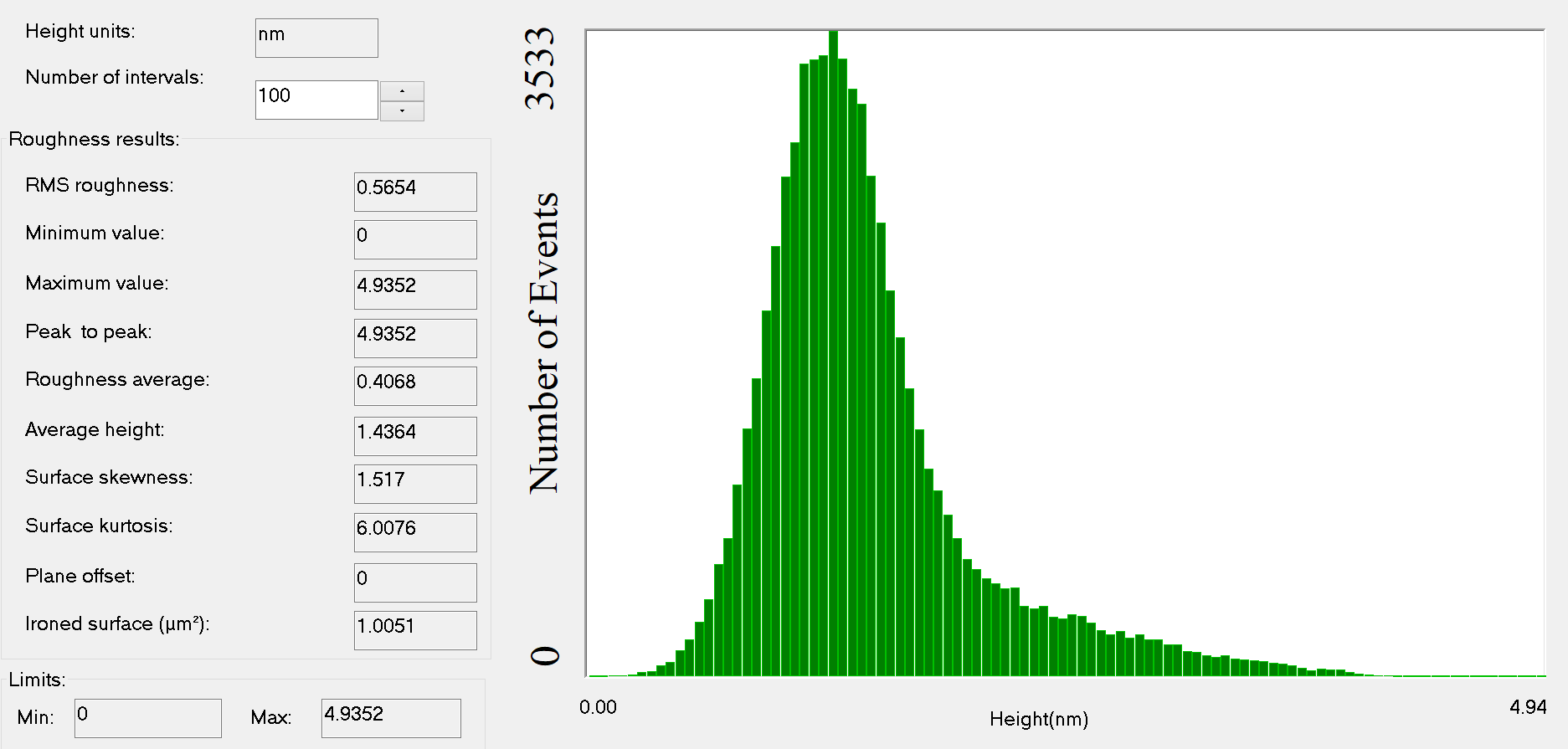


Figure S20: AFM histogram corresponding to sample of figure S19c.

## SEM on polypropylene plastic tubes


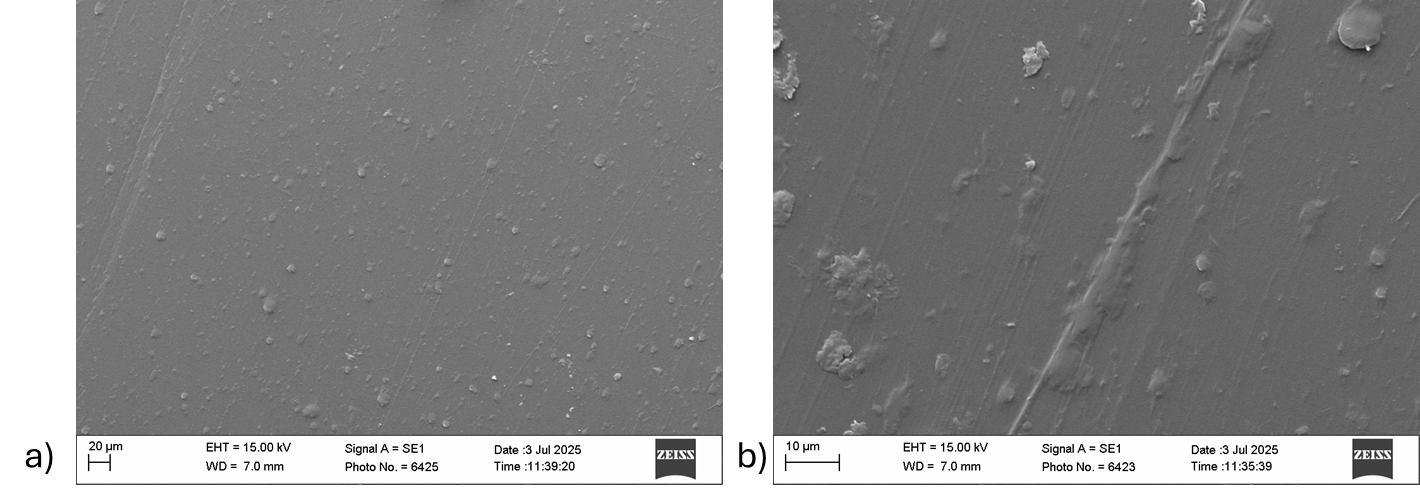


Figure S21: SEM images of PP plastic tubes at a) 500x b) 2500 x. Scale bars are 20 μm and 10 μm, respectively.


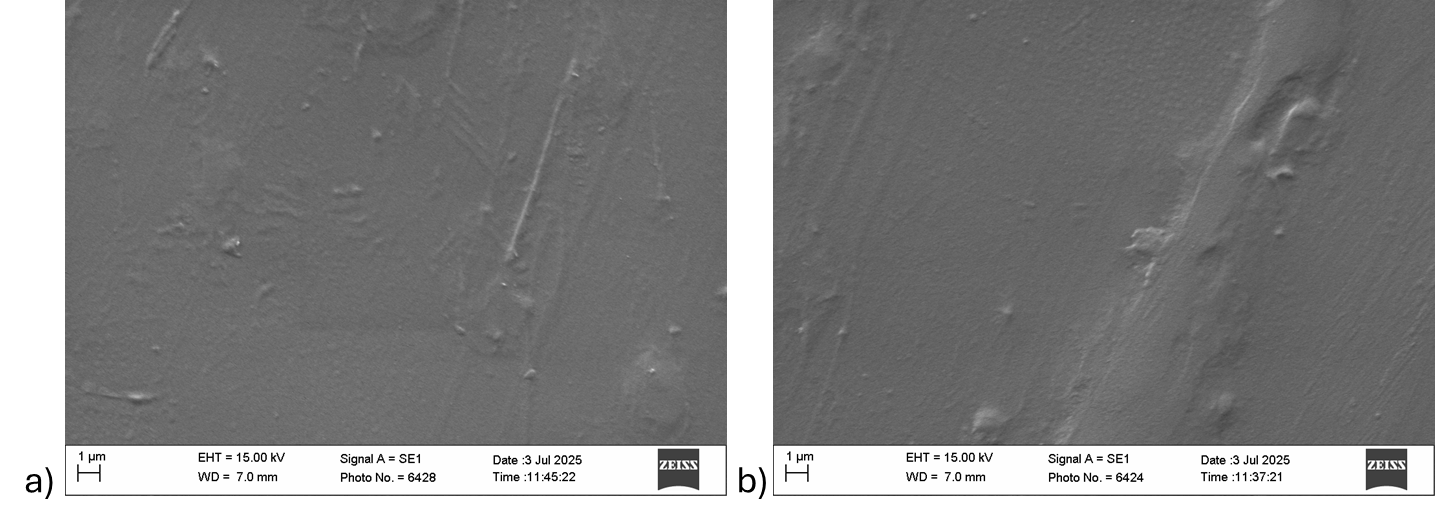


Figure S22: SEM images of PP plastic tubes at 10000x for two different spots a), b). Scale bar is 1 μm.

Figures S21 and S22 show that the plastic tubes do not have a fully homogeneous and flat surface. All images show that the plastic has some regions where the surface protrudes. In figure S22 smaller patterns, of around 100 nm, can be seen, especially on figure S22. This small, similar pattern might be covering a big part of the surface and could be the crystalline parts of the PP structure. SEM images overall saw that the plastic surface is not homogeneous across all the surfaces and some surface roughness is shown. Atomic force microscopy (AFM) could not be performed on this surface because its curvature prevents proper probe contact; the same limitation applies to the glass tubes.
